# Dual PARP/Tankyrase Inhibition Enhances Antitumor Efficacy in PTEN‐Deficient Endometrial Cancer

**DOI:** 10.1111/jcmm.71242

**Published:** 2026-06-12

**Authors:** Sung Wan Kang, Hyerim Eum, Ji‐Young Lee, Min‐Seo Lee, Yong‐Man Kim, Tae Won Kim, Shin‐Wha Lee

**Affiliations:** ^1^ Department of Obstetrics and Gynecology Asan Medical Center, University of Ulsan College of Medicine Seoul Republic of Korea; ^2^ Asan Preclinical Evaluation Center for Cancer TherapeutiX Asan Medical Center Seoul Republic of Korea; ^3^ Department of Biomedical Research Center Asan Medical Center, University of Ulsan College of Medicine Seoul Republic of Korea; ^4^ Department of Oncology Asan Medical Center, University of Ulsan College of Medicine Seoul Republic of Korea

**Keywords:** DNA repair pathway, endometrial cancer, PARP inhibitor, PTEN, tankyrase

## Abstract

Endometrial cancer (EC) incidence continues to rise, underscoring the need for effective therapies beyond BRCA‐mutant disease. Although PTEN loss, a frequent alteration in EC, has been implicated in impaired homologous recombination and increased sensitivity to PARP inhibitors, responses to PARP inhibitor monotherapy remain variable and are often limited by resistance mechanisms in PTEN‐deficient tumours. Here, we show that the dual PARP/tankyrase (TNKS) inhibitor JPI‐547 exerts potent antitumor activity, particularly in PTEN‐deficient Ishikawa tumours. In vitro, JPI‐547 and olaparib more effectively reduced cell survival in PTEN‐deficient cells, and combined treatment with olaparib and the TNKS inhibitor XAV‐939 induced synergistic cytotoxicity with elevated DNA double‐strand breaks. Dual PARP/TNKS inhibition did not further suppress RAD51 but modulated non‐homologous end joining and attenuated Wnt/β‐catenin signalling in a PTEN‐dependent manner. PTEN knockdown further showed enhanced vulnerability to combined targeting. These findings show that JPI‐547 enhances antitumor efficacy in PTEN‐deficient EC by disrupting DNA repair pathways and Wnt signalling, supporting dual PARP/TNKS inhibition as a potential therapeutic strategy and providing a rationale for further clinical evaluation.

## 1 Introduction

Endometrial cancer (EC) is the most common gynecologic malignancy in developed countries, with its incidence continuing to rise due to aging populations and increasing obesity rates. While early‐stage endometrioid endometrial cancer generally has a favourable prognosis, advanced or recurrent EC remains challenging to treat and is often associated with poor clinical outcomes [1, 2, 3].

Poly (ADP‐ribose) polymerase (PARP) inhibitors have demonstrated therapeutic efficacy in tumours with homologous recombination deficiency (HRD), such as those harbouring BRCA1/2 mutations, by exploiting synthetic lethality [4, 5]. These agents trap PARP1 on damaged DNA, converting single‐strand breaks into cytotoxic double‐strand breaks in HR‐deficient cells [6]. PARP inhibitors have also shown efficacy in tumours without BRCA mutations, and their clinical applications continue to expand [7, 8]. Therefore, reliable biomarkers of non‐BRCA HRD are needed to predict therapeutic responses to PARP inhibition.

One of the most frequently altered genes in EC is *PTEN* (phosphatase and tensin homologue), a tumour suppressor that plays a crucial role in maintaining chromosomal stability and facilitating DNA damage repair through the regulation of homologous recombination [9, 10, 11]. Previous studies using EC models have demonstrated that PTEN loss is associated with increased sensitivity to PARP inhibition; however, responses to single‐agent PARP inhibitors are variable and often limited by resistance mechanisms [12, 13, 14]. Consequently, combination strategies involving PARP inhibitors and therapeutic targeting of the PTEN‐PI3K‐AKT signalling pathway or BCL‐2‐associated death promoter (BAD) phosphorylation have been investigated in PTEN‐deficient ECs, showing enhanced antitumor activity [10, 13, 14, 15, 16].

Tankyrase 1 and 2 (TNKS1/2), also known as PARP5a and PARP5b, are members of the PARP family that regulate critical pathways, including Wnt/β‐catenin signalling, telomere homeostasis and DNA repair [17, 18]. Inhibition of TNKS has been shown to suppress tumour progression by disrupting the Wnt/β‐catenin signalling pathway [19, 20]. Moreover, TNKS promotes HR by facilitating RAD51 recruitment to DNA double‐strand breaks (DSBs) and activating DNA damage checkpoint signalling [21]. The catalytic domains of TNKS1/2 share high sequence homology with that of PARP1 [22]. Several PARP inhibitors, such as talazoparib and JPI‐547, potently inhibit PARP1 and also target TNKS1/2. Notably, JPI‐547 exhibits stronger inhibitory activity against TNKS1/2 while maintaining comparable potency against PARP1/2 compared to conventional PARP inhibitors. JPI‐547 was developed to simultaneously target both DNA repair and Wnt signalling pathways [22, 23, 24].

In this study, we evaluated the antitumor efficacy of PARP inhibitors, including JPI‐547, in EC xenograft models harbouring either wild‐type or mutant PTEN. Additionally, we investigated the therapeutic potential of dual inhibition of PARP1/2 and TNKS1/2 in PTEN‐deficient EC using a series of in vitro and in vivo analyses.

## 2 Materials and Methods

### 2.1 Cell Lines

The Hec‐1A and Hec‐1B cell lines were obtained from the American Type Culture Collection (ATCC, Manassas, VA, USA). The Ishikawa cell line was purchased from Sigma‐Aldrich (Saint Louis, MO, USA). All cell lines were cultured in RPMI‐1640 medium supplemented with 10% fetal bovine serum and 1% antibiotic‐antimycotic solution at 37°C, and routinely tested for mycoplasma contamination. Cell line authentication was confirmed within the past 3 years using short tandem repeat profiling.

### 2.2 Mice

Female BALB/c slc‐nu/nu mice (6 weeks old) were purchased from JA BIO (Suwon, Korea). All animals were maintained in a specific pathogen‐free facility at the Disease Animal Research Center of Asan Medical Center (Seoul, Korea). Sterilised food and water were provided ad libitum. All animal experiments were approved by the Institutional Animal Care and Use Committee (IACUC) of the Asan Institute for Life Sciences, Asan Medical Center, and were carried out in accordance with the approved protocols and relevant regulations (approval ID: 2023‐20‐247). This study also complied with the ARRIVE guidelines where applicable.

### 2.3 In Vivo Experiments

Subcutaneous xenograft models were established by injecting 2 × 10^6^ Hec‐1A or Ishikawa cells mixed with 50% Matrigel into the right hind limb of each mouse. No formal sample size calculation was performed. Group size was chosen based on commonly used numbers in similar xenograft studies. When tumour volumes reached approximately 150 mm^3^ for the Hec‐1A model or 110 mm^3^ for the Ishikawa model, mice were randomly assigned to treatment groups: vehicle control, olaparib (50 mg/kg), niraparib (50 mg/kg), talazoparib (0.15 mg/kg) or JPI‐547 (50 mg/kg), with eight or six mice per group. All drugs were administered orally once daily. Mice were treated for five consecutive days, followed by a 2‐day rest period, repeated for four to five cycles. Tumour growth was regularly measured using callipers, and tumour volume data were used to calculate tumour growth inhibition. No blinding was performed. Mice were euthanised with CO_2_ gas 2 h after the final dose, and tumour tissues were collected for analyses. No animals or data points were excluded from the analysis.

### 2.4 Histopathological and Immunostaining Analyses of Tumour Tissues

Following the final measurement of tumour volume, all tumour‐bearing mice were humanely euthanised in accordance with the approved experimental protocol. Subcutaneous tumour tissues were collected, fixed in formalin and embedded in paraffin for histopathological analysis. Tissue sections were stained with haematoxylin and eosin (H&E) for morphological assessment. Apoptotic cells were detected using the in situ cell death detection kit POD (Roche, Basel, Switzerland), following the manufacturer's instructions.

Immunohistochemistry (IHC) and immunofluorescence (IF) staining were performed as previously described [25]. Briefly, antigen retrieval was carried out by heating formalin‐fixed, paraffin‐embedded (FFPE) sections in Tris‐EDTA buffer (Vector Laboratories, Newark, CA, USA). Subsequently, endogenous peroxidase activity was quenched using BLOXALL blocking solution (Vector Laboratories), and non‐specific binding was minimised by incubating the sections with 2.5% normal goat serum (Vector Laboratories).

For IHC, sections were incubated overnight at 4°C with an anti‐Ki67 antibody (SP6, Abcam, Cambridge, MA, USA), followed by incubation with biotinylated rabbit IgG (Abcam). For IF, sections were incubated for 2 h at room temperature (RT) with fluorophore‐conjugated antibodies: Alexa Fluor 488 anti‐Rad51 (Santa Cruz Biotechnology, Dallas, TX, USA) and Alexa Fluor 647 anti‐γ‐H2AX (Abcam). IHC detection was performed using avidin‐biotin complex reagents (Vector Laboratories), developed with DAB substrate (Vector Laboratories) and counterstained with 1% methyl green (R&D Systems, Minneapolis, MN, USA). Slides were mounted using appropriate media for IHC (Thermo Fisher Scientific, Waltham, MA, USA) or IF (with DAPI, Vector Laboratories). Representative images of H&E, IHC and IF‐stained sections were acquired using the VS200 digital slide scanner (Olympus, Tokyo, Japan).

Quantitative analysis of γ‐H2AX and RAD51 staining was performed using ImageJ software. For each tumour, three to four randomly selected non‐necrotic fields were analysed, with three tumours included per treatment group. γ‐H2AX‐positive cells were defined as tumour cells exhibiting nuclear γ‐H2AX staining above background levels, and the number of γ‐H2AX‐positive cells was counted per field. RAD51 expression was quantified as the percentage of RAD51‐positive area relative to the total tumour area. After excluding necrotic and non‐tumour regions, a uniform intensity threshold was applied across all images to identify RAD51‐positive signal.

### 2.5 Cell Viability Assay

Cell viability was assessed by quantifying intracellular ATP levels using the CellTiter‐Glo luminescent cell viability assay (Promega, Madison, WI, USA). Cells were seeded into 96‐well plates at a density of 2000 cells/well for Hec‐1A and Hec‐1B cells, and 1000 cells/well for Ishikawa cells. After 24 h, cells were treated with JPI‐547, olaparib, XAV‐939 or a combination of olaparib and XAV‐939 at serially diluted concentrations. Following 5 days of treatment, the CellTiter‐Glo assay was performed according to the manufacturer's instructions. Luminescence was measured using a VICTOR multilabel plate reader (PerkinElmer, Waltham, MA, USA). Dose–response curves and IC_50_ values were calculated using GraphPad Prism (GraphPad Software, La Jolla, CA, USA). Combined effects were determined by the Chou‐Talalay method using CompuSyn software [26]. Combination index (CI) values were interpreted as follows: synergism (CI < 1), additivity (CI = 1) and antagonism (CI > 1).

### 2.6 Clonogenic Assay

Cells were seeded in 6‐well plates in triplicate at a density of 1000 cells/well and treated with JPI‐547, olaparib and XAV‐939 individually at serially diluted concentrations, or with a combination of olaparib and XAV‐939. Following a 72‐h incubation at 37°C in a humidified atmosphere with 5% CO_2_, fresh medium was replaced every 3 days. Cells were cultured for 10–14 days until colonies were visibly formed. Colonies were fixed and stained with 0.5% (w/v) crystal violet in 20% (v/v) methanol for 1 h at RT, followed by two washes with PBS. Images were acquired using a GelCount colony counter (Oxford Optronix, Abingdon, UK), and colony numbers were quantified using ImageJ software. Dose–response curves and IC_50_ values were calculated using GraphPad Prism.

### 2.7 Comet Assay

A neutral comet assay was performed to quantitatively evaluate DSBs in compound‐treated cells using the CometAssay Kit (Trevigen, Gaithersburg, MD, USA), following the manufacturer's protocol. Briefly, 1 × 10^5^ cells were resuspended in 50 μL of PBS, and 20 μL of the suspension was mixed with 200 μL of 1% low‐melting‐point agarose. Then, 50 μL of the mixture was layered onto a CometSlide (Trevigen) and allowed to solidify at 4°C for 10 min. Slides were incubated in cold lysis buffer (Trevigen) for 1 h, followed by equilibration in cold Tris/Borate/EDTA (TBE) buffer for 30 min. Electrophoresis was conducted for 30 min at 18 V using a Mupid‐One system in cold TBE buffer. Subsequently, DNA was precipitated with 1 M ammonium acetate in 95% ethanol for 30 min at RT, followed by immersion in 70% ethanol for an additional 30 min and air‐dried overnight. DNA was stained with SYBR Gold (Invitrogen, Waltham, MA, USA) for 30 min, and images were captured using the EVOS FL Auto Imaging System (Life Technologies, Carlsbad, CA, USA). Image analysis was performed using CometScore 2.0 software, and DNA damage was quantified by calculating the percentage of tail DNA and tail moment.

### 2.8 siRNA Transfection

PTEN‐specific siRNA and non‐targeting control siRNA were obtained from Santa Cruz Biotechnology. Cells (2 × 10^5^ per well) were seeded in 6‐well plates and incubated at 37°C until they reached approximately 70% confluence. Transfection was performed using a siRNA transfection reagent (Santa Cruz Biotechnology) according to the manufacturer's instructions. Six hours after transfection, an equal volume of 2× complete growth medium was added directly to the wells without washing the cells. The cells were then incubated for an additional 24 h, after which the medium was replaced with fresh complete growth medium. Following a further 24‐h incubation, the transfected cells were harvested by trypsinisation and replated for subsequent assays. Drug treatments were administered approximately 16 h after seeding.

### 2.9 Western Blot Analysis

Hec‐1A and Ishikawa cells were lysed in RIPA buffer (Sigma‐Aldrich) supplemented with cOmplete Protease Inhibitor Cocktail (Roche) and PhosSTOP phosphatase inhibitor (Roche). Protein concentrations were determined using the Pierce BCA Protein Assay Kit (Thermo Fisher Scientific). Protein lysates were separated by SDS‐PAGE (6%–15%) and subsequently transferred to polyvinylidene difluoride (PVDF) membranes (Merck Millipore, Burlington, MA, USA).

After blocking the membranes with 5% skim milk in TBST for 1 h at RT, they were incubated overnight at 4°C with the respective primary antibodies against DNA‐dependent protein kinase catalytic subunit (DNA‐PKcs; Y393, Abcam), Ku70 (polyclonal antibody, Abcam), PTEN (EPR4408‐76, Abcam), β‐catenin (E247, Abcam), phospho‐β‐catenin (polyclonal antibody, Cell Signaling Technology, MA, USA), Axin1 (C76H11, Cell Signaling Technology), Axin2 (polyclonal antibody, Abcam) or β‐actin (C4, Santa Cruz Biotechnology), all diluted in the blocking buffer. The following day, the membranes were incubated with horseradish peroxidase‐conjugated goat anti‐rabbit or anti‐mouse secondary antibodies (Santa Cruz Biotechnology), as appropriate. After 2 h at RT, signals were developed using an enhanced chemiluminescence solution (Thermo Fisher Scientific). Band intensities were quantified and analysed with the ImageQuant LAS 4000 imager (GE Healthcare, Buckinghamshire, UK).

### 2.10 Statistical Analysis

All in vitro experiments were conducted in three independent experiments. The data are presented as the mean ± standard deviation (SD). Statistical analysis was performed using GraphPad Prism software with unpaired two‐tailed Student's *t*‐tests used for pairwise comparisons and one‐way analysis of variance (ANOVA) followed by Tukey's or Bonferroni's post hoc test used for multiple comparisons. A *p* value of less than 0.05 was considered statistically significant.

### 2.11 Supplementary Materials and Methods

Additional experimental procedures and methodological details are provided in the Supplementary methods.

## 3 Results

### 3.1 Comparison of the Growth Inhibition Effects of PARP Inhibitors in Hec‐1A and Ishikawa Xenografts

Given that PTEN loss impairs HR and sensitises tumour cells to PARP inhibition [14], we selected two representative EC cell lines with distinct PTEN statuses. Hec‐1A retains wild‐type PTEN, whereas Ishikawa harbours PTEN mutations. To examine the in vivo efficacy of PARP inhibitors, including olaparib, niraparib, talazoparib and JPI‐547, xenograft models using Hec‐1A and Ishikawa cells were established, and mice were treated accordingly.

**FIGURE 1:**
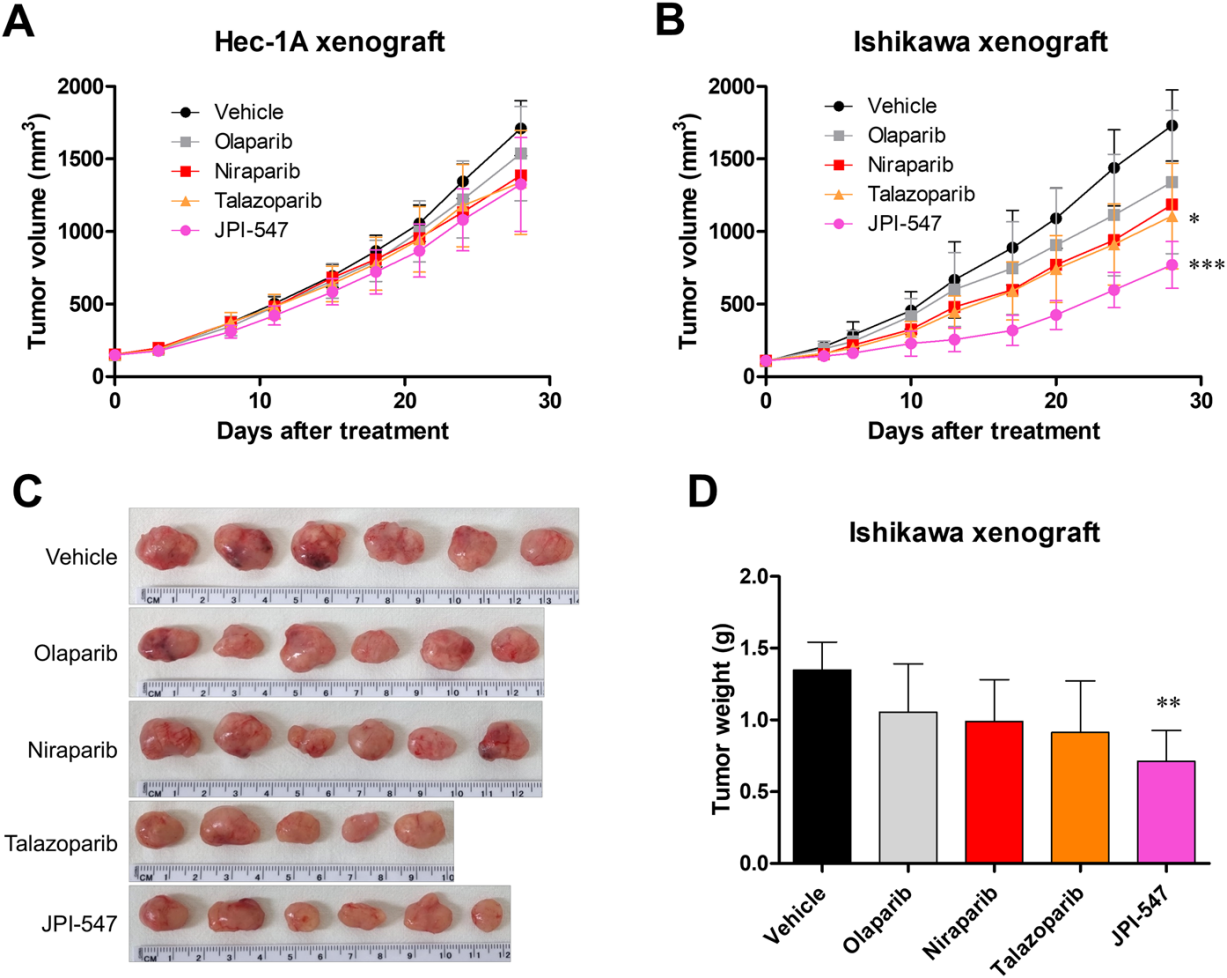
Differential response to PARP inhibitors in PTEN wild‐type (Hec‐1A) and PTEN‐mutant (Ishikawa) EC xenografts. (A, B) Tumour growth curves for Hec‐1A (A) and Ishikawa (B) xenograft models treated with vehicle, olaparib, niraparib, talazoparib or JPI‐547. Hec‐1A or Ishikawa cells were injected subcutaneously into female BALB/c nude mice. When tumours reached a volume of approximately 110–150 mm^3^, mice were grouped and treated. Tumour volumes were measured twice weekly using callipers. (C) Representative images of excised tumours at the study endpoint in the Ishikawa model. (D) Final tumour weights were measured for each treatment group. Data are presented as mean ± SD. (One‐way ANOVA followed by Bonferroni's post hoc test, *p < 0.05, **p < 0.01, ***p < 0.001.) Asterisks (*) indicate statistical significance compared with the vehicle control.

In the Hec‐1A xenograft model, slight tumour growth inhibition was observed in each treatment group; however, these differences did not reach statistical significance compared to the control group (Figure 1A). In contrast, PARP inhibitors with JPI‐547 and talazoparib showed significant tumour growth inhibition in the Ishikawa CDX model (Figure 1B). Notably, JPI‐547 demonstrated superior tumour growth inhibitory efficacy compared to the other PARP inhibitors in this experimental setting. All treatments were well tolerated, and although minor body weight decreases were observed with niraparib and talazoparib, these changes were transient and not statistically significant in either model (Figure S1).

Consistent with tumour growth curves, significant decreases in tumour weight were observed following treatment with JPI‐547. In contrast, treatment with olaparib, niraparib or talazoparib did not result in a statistically significant reduction in tumour weight (Figure 1C,D). Collectively, these findings indicate that JPI‐547 exerts potent antitumor effects in the PTEN‐deficient Ishikawa model but shows limited efficacy in the PTEN‐proficient Hec‐1A xenograft model.

### 3.2 PARP Inhibitors Reduce the Proliferation of Ishikawa Xenograft Tumours and Induce Apoptosis

**FIGURE 2:**
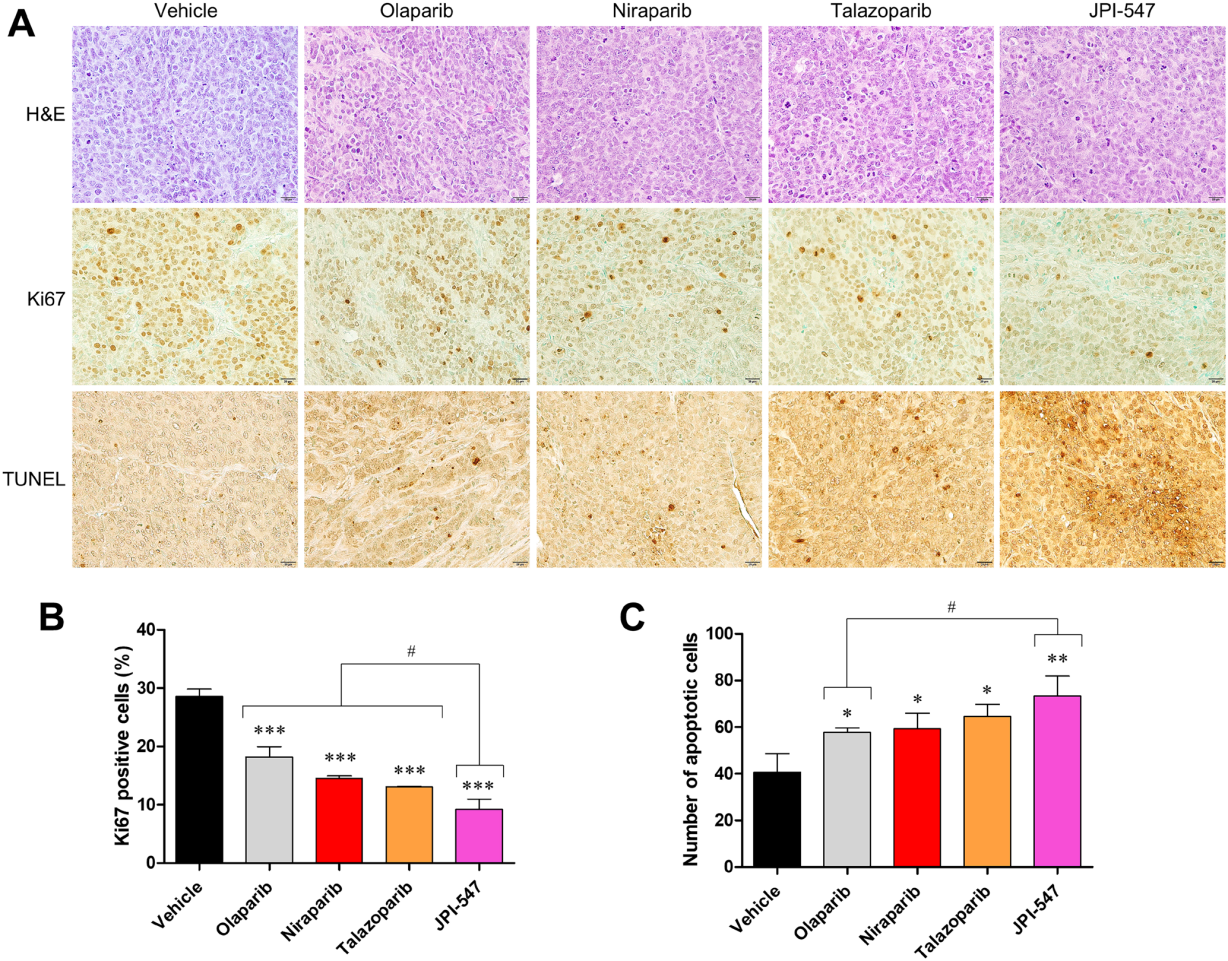
PARP inhibitors reduce tumour proliferation and induce apoptosis in Ishikawa xenograft tumours. (A) Representative images of excised Ishikawa tumours treated with vehicle, olaparib, niraparib, talazoparib or JPI‐547, stained for morphology (H&E, top), Ki‐67 IHC (middle) and TUNEL assay (bottom). Original magnification, ×200; Scale bar, 20 μm. (B) Quantification of Ki‐67‐positive proliferating cells and (C) TUNEL‐positive apoptotic cells demonstrates a significant reduction in cell proliferation and an increase in apoptosis in all PARPi‐treated groups compared to the vehicle control group. Data are presented as mean ± SD. Statistical significance was determined using an unpaired two‐tailed Student's *t*‐test. *p < 0.05, **p < 0.01 and ***p < 0.001 versus vehicle. #*p* < 0.05 for comparisons indicated by brackets.

To evaluate proliferation and apoptosis associated with tumour growth inhibition by PARP inhibitors, including JPI‐547, IHC assays were conducted on Ishikawa xenograft tumours (Figure 2A). IHC analysis of Ki67 revealed a significant decrease in proliferation across all PARP inhibitor‐treated groups compared to the vehicle control group, confirming the effective suppression of tumour cell proliferation by JPI‐547 and other PARP inhibitors. Notably, JPI‐547 treatment resulted in a significantly greater reduction in proliferative cells compared to other PARP inhibitors (Figure 2B). Furthermore, TUNEL assays demonstrated increased apoptosis in all PARP inhibitor‐treated groups compared to the vehicle control group, with a significant difference observed between JPI‐547 and olaparib (Figure 2C). These findings indicate that JPI‐547 effectively inhibits proliferation and induces apoptosis in PTEN‐mutant Ishikawa tumours.

### 3.3 Dual Inhibition of PARP1/2 and TNKS1/2 via JPI‐547 Treatment Increases DNA Damage in Ishikawa Xenograft Tumours Without Significantly Affecting RAD51 Expression

**FIGURE 3:**
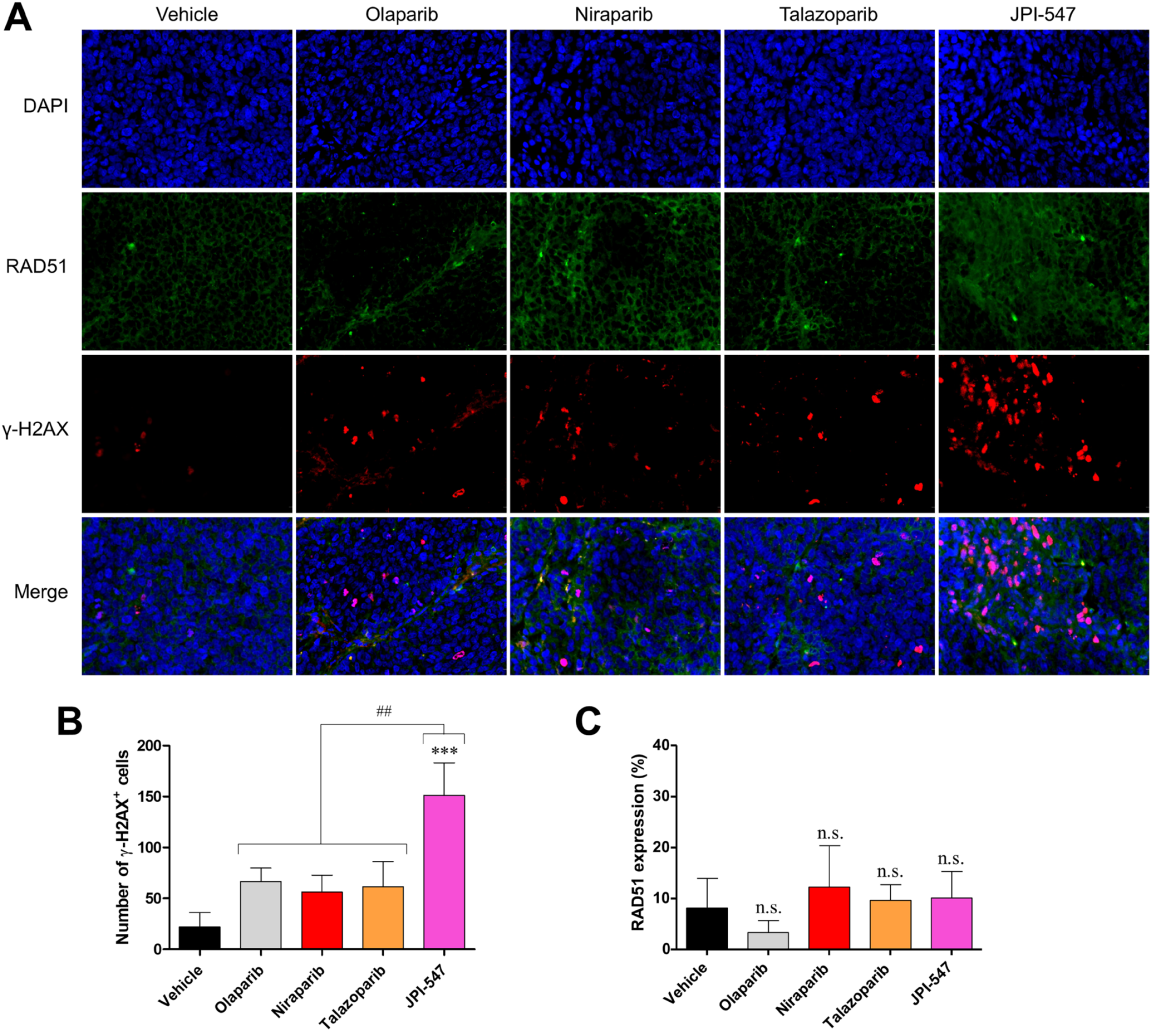
JPI‐547 enhances γ‐H2AX levels without affecting RAD51 expression in Ishikawa xenograft tumours. (A) Representative IF images of Ishikawa xenograft tumours treated with vehicle, olaparib, niraparib, talazoparib or JPI‐547. Tumour sections were stained for γ‐H2AX (red), RAD51 (green) and nuclei (DAPI, blue). Quantitative analysis of (B) γ‐H2AX and (C) RAD51 staining reveals a significant increase in γ‐H2AX‐positive cells, while RAD51 expression remains unchanged (unpaired two‐tailed Student's *t*‐test, ***p < 0.001, ##*p* < 0.01, n.s. = not significant). Asterisks (*) indicate statistical significance compared with the vehicle control.

Given that JPI‐547 is a dual inhibitor targeting both PARP1/2 and TNKS1/2, and that PTEN loss has been variably associated with RAD51 expression [10, 13, 27], we examined γ‐H2AX and RAD51 in PTEN‐deficient tumours to assess DNA damage and RAD51 levels. To this end, we performed IF staining for γ‐H2AX and RAD51 in Ishikawa xenograft tumours (Figure 3A). The number of γ‐H2AX‐positive cells was elevated in all treatment groups compared to the vehicle control group (Figure 3B). Notably, JPI‐547 treatment resulted in a significantly higher number of γ‐H2AX‐positive cells than other PARP inhibitor‐treated groups, indicating a pronounced accumulation of DNA damage.

In contrast, RAD51 expression levels did not exhibit significant differences across treatment groups (Figure 3C). Although the olaparib group showed a slight decrease in RAD51 expression relative to the vehicle control, this difference was not statistically significant. These findings suggest that JPI‐547 induces DNA damage in PTEN‐deficient tumours without detectable changes in RAD51 expression, suggesting that the observed DNA damage is not associated with changes in RAD51 expression.

### 3.4 The Cytotoxic Effects of JPI‐547 and Olaparib Are More Pronounced in PTEN‐Deficient Ishikawa Cells Than in PTEN‐Wild Type EC Cells

To further elucidate the antitumor effects of combined PARP1/2 and TNKS1/2 inhibition observed in the EC xenograft model, Hec‐1A, Hec‐1B (PTEN wild‐type), and Ishikawa (PTEN‐deficient) cells were treated with JPI‐547, olaparib or the TNKS1/2 inhibitor XAV‐939, followed by evaluation of cell viability and clonogenic survival.

**FIGURE 4:**
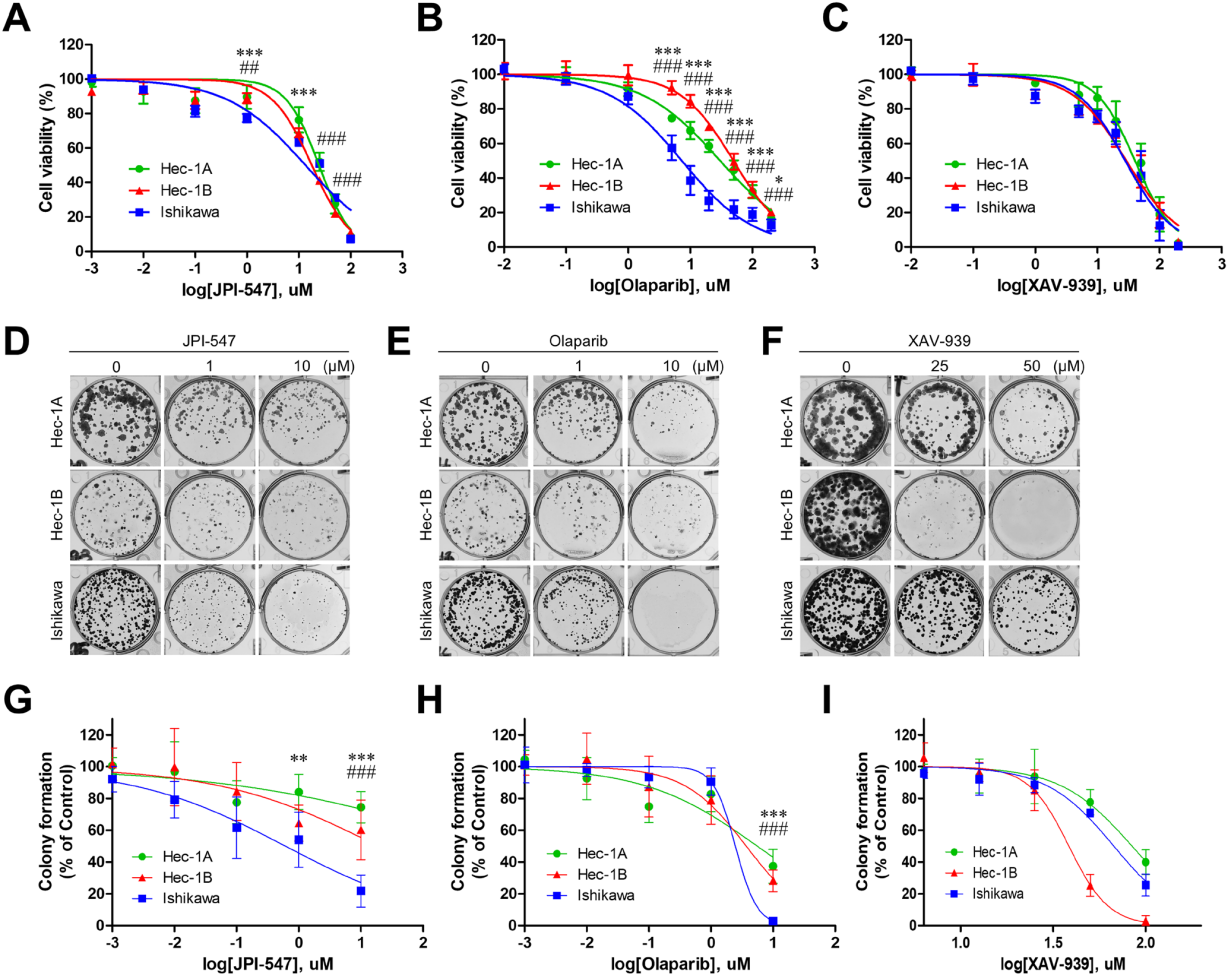
JPI‐547 and olaparib show higher anti‐proliferative sensitivity in Ishikawa EC cells. (A–C) Cell viability assays in Hec‐1A, Hec‐1B and Ishikawa cells exposed to increasing concentrations of olaparib, XAV‐939 or JPI‐547 for 120 h. (D–F) Representative images from clonogenic assays performed on Hec‐1A, Hec‐1B and Ishikawa cells following treatment with olaparib, XAV‐939 or JPI‐547. Cells were incubated for 7–12 days to allow colony formation after treatment and subsequently stained with crystal violet for visualisation. (G–I) Colony formation was quantified after treatment with increasing concentrations of each compound to assess dose‐dependent effects. Data are presented as mean ± SD. Statistical significance was determined using one‐way ANOVA followed by Tukey's post hoc test. *p < 0.05, **p < 0.01, ***p < 0.001 versus Hec‐1A; ##*p* < 0.01, ###*p* < 0.001 versus Hec‐1B.

Ishikawa cells exhibited higher sensitivity to both JPI‐547 and olaparib compared to Hec‐1A and Hec‐1B cells in the viability assay, but not to XAV‐939 treatment (Figure 4A–C and Figures S2 and S3). While olaparib demonstrated more pronounced differences in sensitivity between Ishikawa and Hec‐1A/B cells, JPI‐547 exhibited minimal variation in short‐term viability across all three cell lines.

Long‐term clonogenic survival following drug treatment was markedly reduced by JPI‐547 in Ishikawa cells. In contrast, olaparib showed a less pronounced suppression of clonogenic potential in Ishikawa cells compared to Hec‐1A and Hec‐1B cells. Treatment with XAV‐939 alone had limited effects across all cell lines (Figure 4D–I and Figure S4). Together, these data indicate that both JPI‐547 and olaparib are effective in PTEN‐deficient Ishikawa cells, with JPI‐547's enhanced activity being more evident in the context of long‐term survival.

### 3.5 Combined Treatment With Olaparib and XAV‐939 Results in Synergistic Eeffects in Ishikawa Cells, and PTEN Knockdown in Hec‐1A Cells Increases Their Sensitivity to These Inhibitors

**FIGURE 5:**
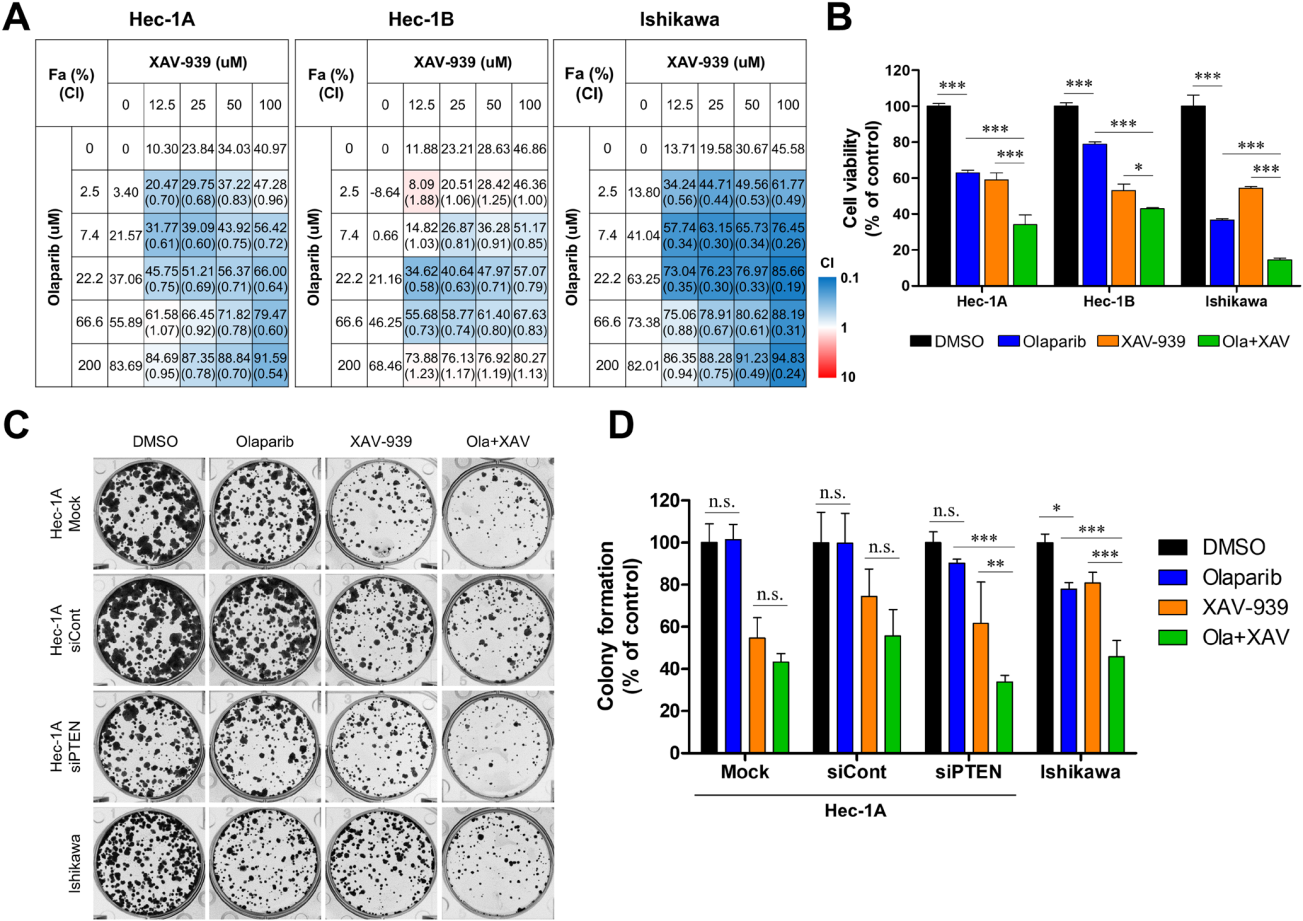
Olaparib and XAV‐939 synergistically inhibit cell growth and clonogenic survival more effectively in PTEN‐deficient EC cells than in PTEN‐proficient EC cells. (A) Hec‐1A, Hec‐1B and Ishikawa EC cells were treated with olaparib and XAV‐939 alone or in combination at the indicated concentrations for 120 h. Cell viability was assessed using the luminescent CellTiter‐Glo assay. CI values were calculated using CompuSyn software based on the Chou‐Talalay method to evaluate drug synergy. (B) Representative cell viability data from the combination analysis shown in panel (A). Hec‐1A, Hec‐1B and Ishikawa cells were treated with olaparib (22.2 μM), XAV‐939 (100 μM) or the combination. Data are presented as mean ± SD from three independent experiments. Statistical significance was determined using one‐way ANOVA followed by Bonferroni's post hoc test (*p < 0.05, ***p < 0.001). (C) Representative images of clonogenic assays in Hec‐1A and Ishikawa cells treated with DMSO, olaparib (2 μM), XAV‐939 (50 μM) or the combination of both agents. Hec‐1A cells were transfected with PTEN siRNA (siPTEN) or control siRNA (siCont) and seeded into 6‐well plates. Following drug treatment, cells were cultured for an additional 7 to 12 days and stained with crystal violet. (D) Quantitative analysis confirmed the enhanced inhibitory effect of the olaparib and XAV‐939 combination on cell survival. Statistical significance was determined using an unpaired two‐tailed Student's *t*‐test (*p < 0.05, **p < 0.01, ***p < 0.001, n.s. = not significant).

Next, to evaluate the potential therapeutic benefit of combined PARP and TNKS inhibition in EC, we treated Hec‐1A, Hec‐1B and Ishikawa cells with increasing concentrations of olaparib and XAV‐939, both individually and in combination. Cell viability was assessed, and CI values were calculated to determine drug interaction patterns (Figure 5A,B and Table S1). In Hec‐1A and Hec‐1B cells, the combination treatment with olaparib and XAV‐939 predominantly resulted in moderate synergistic effects across multiple concentration ranges. In Ishikawa cells, most drug combinations yielded CI values substantially below 1.0 (range: 0.19–0.56), indicating strong synergy. This synergistic effect was most pronounced at relevant concentrations of olaparib (7.4–22.2 μM) combined with XAV‐939 (25–100 μM).

We further investigated whether PTEN status affects sensitivity to these inhibitors using clonogenic assays (Figure 5C,D). To determine whether PTEN loss influences the observed synergistic effect, PTEN expression was silenced in Hec‐1A cells via siRNA‐mediated knockdown. Hec‐1A cells with PTEN silencing exhibited significantly increased sensitivity to the combination of olaparib and XAV‐939 compared to cells transfected with control siRNA. Similarly, in Ishikawa cells, the combination treatment markedly reduced cell survival compared to either agent alone (Figure 5C,D). These results suggest that PTEN deficiency enhances the synergistic anticancer effect elicited by combined inhibition of PARP and TNKS.

### 3.6 Combined PARP and TNKS Inhibition Exacerbates DNA Damage in Ishikawa and PTEN‐Knockdown Hec‐1A Cells

**FIGURE 6:**
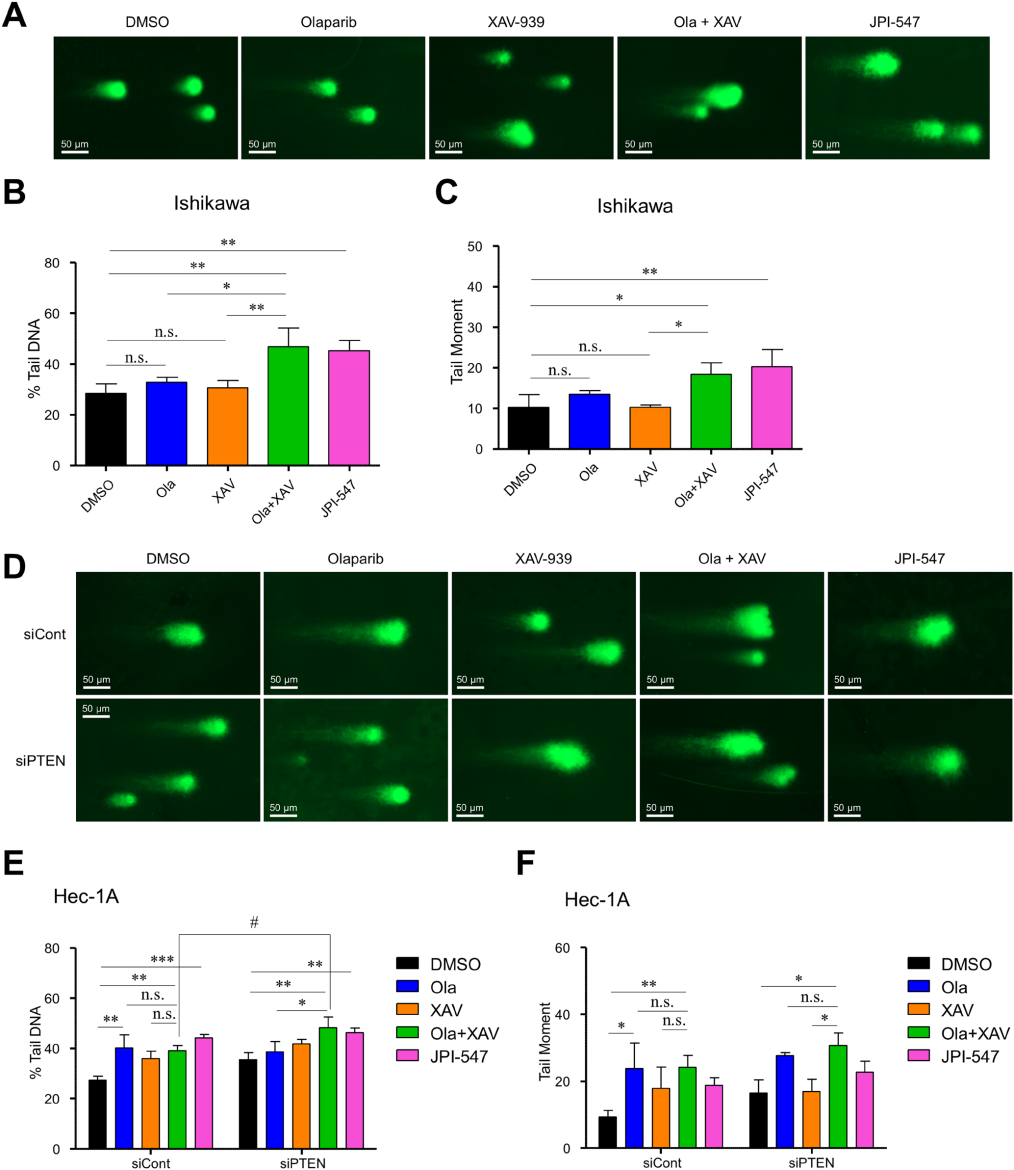
Comet assay reveals differential DNA damage following PARP and TNKS inhibition in PTEN‐deficient and proficient EC cells. (A) Representative comet assay images of Ishikawa cells following a 48‐h treatment with DMSO, olaparib (10 μM), XAV‐939 (30 μM), their combination or JPI‐547 (10 μM). DNA fragmentation was visualised as comet tails by fluorescence microscopy. Increased tail length and fluorescence intensity indicate elevated levels of DSBs. Quantification of DNA damage in Ishikawa cells, shown as percent tail DNA (B) and tail moment (C), both indicative of the extent of DNA damage. (D) Representative comet images of Hec‐1A cells subjected to PTEN knockdown and treated under the same drug conditions. Quantitative analysis of DNA damage in siPTEN and siControl Hec‐1A cells, measured as percent tail DNA (E) and tail moment (F). Statistical significance was determined by one‐way ANOVA followed by Tukey's post hoc test (*p < 0.05, **p < 0.01, ***p < 0.001, #*p* < 0.05, n.s. = not significant).

To elucidate the molecular mechanisms underlying the effects of dual PARP and TNKS inhibition, comet assays were conducted to assess the extent of DNA damage in EC cells. In Ishikawa cells, co‐treatment with olaparib and XAV‐939 led to a marked increase in comet tail length and intensity, indicating enhanced accumulation of DSBs compared to monotherapy. Similarly, treatment with the dual PARP/TNKS inhibitor JPI‐547 significantly elevated DNA damage levels (Figure 6A–C). In Hec‐1A cells, PTEN knockdown tended to increase DNA damage (Figure 6D–F). Notably, in PTEN knockdown Hec‐1A cells, co‐treatment with olaparib and XAV‐939 further elevated tail DNA levels compared to single‐agent treatments, whereas this effect was not observed in PTEN‐proficient Hec‐1A cells (Figure 6D,E).

To further assess the DNA damage response, we evaluated γ‐H2AX and RAD51 in EC cells (Figure S5A–C). The number of γ‐H2AX‐positive cells was significantly increased following olaparib‐containing treatments or JPI‐547 treatment in both Hec‐1A and Ishikawa cells. However, co‐treatment with olaparib and XAV‐939 did not result in an additional increase in γ‐H2AX levels compared to olaparib alone. Consistent with the comet assay findings, PTEN knockdown Hec‐1A cells exhibited an overall trend toward increased DNA damage across treatment conditions, as reflected by elevated γ‐H2AX levels, with statistical significance observed only following olaparib monotherapy (Figure S5B).

In Ishikawa cells, nuclear RAD51 signal was increased following combined treatment with olaparib and XAV‐939 or treatment with JPI‐547, with only the combination reaching statistical significance. In Hec‐1A cells, PTEN knockdown significantly increased the proportion of cells positive for nuclear RAD51 signal. However, additional enhancement of RAD51 signal following dual PARP/TNKS inhibition was not observed in PTEN knockdown Hec‐1A cells (Figure S5C).

### 3.7 Combined PARP and TNKS Inhibition Alters NHEJ and Wnt/β‐Catenin Signalling Components in EC Cells in a PTEN‐Dependent Manner

**FIGURE 7:**
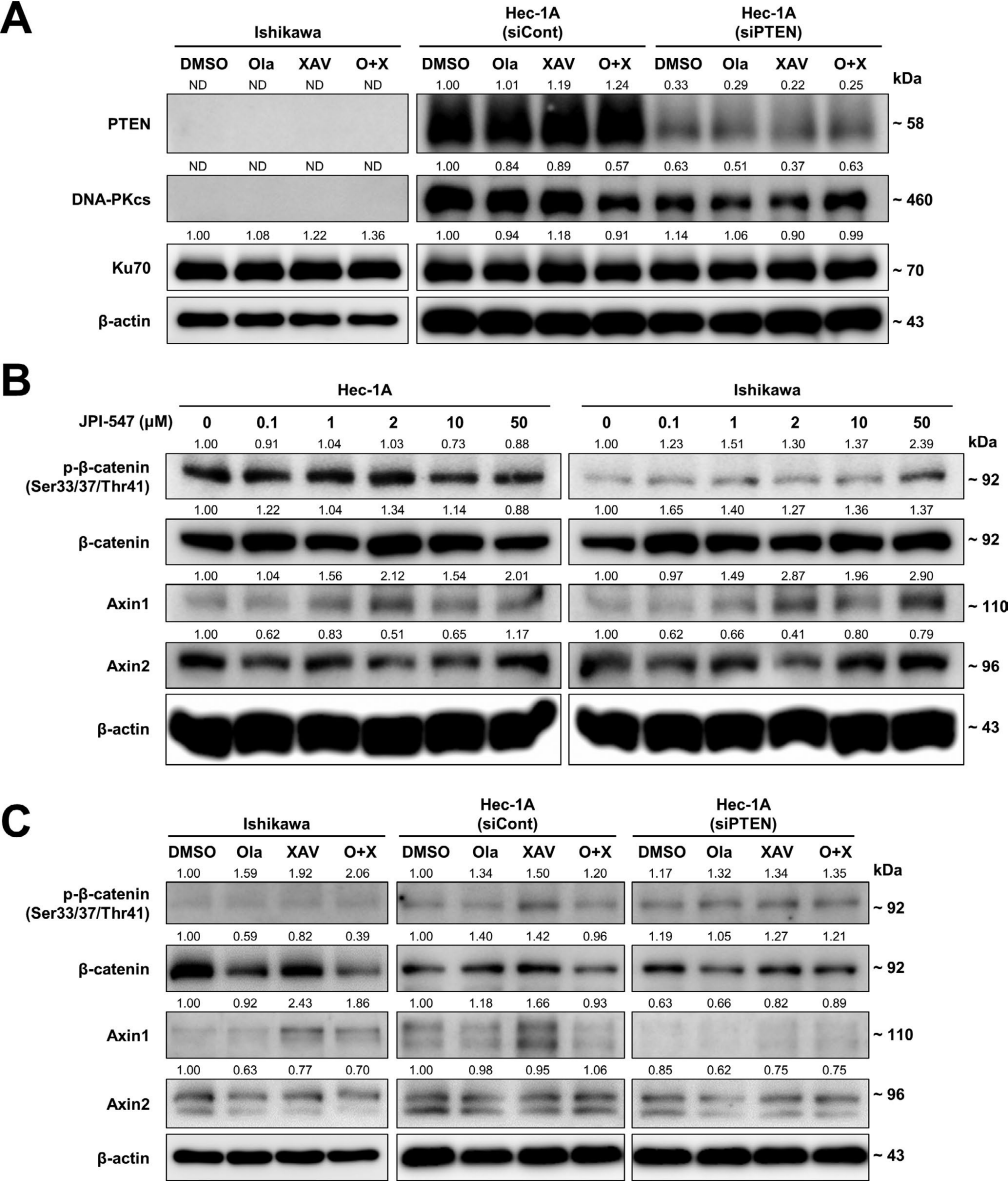
Impact of combined PARP/TNKS inhibition on NHEJ and Wnt signalling pathway proteins in EC cells. (A) Representative immunoblot analysis of DNA‐PKcs and Ku70, key proteins involved in the NHEJ pathway, in Ishikawa and Hec‐1A cells transfected with siPTEN or control siRNA (siCont), following 24‐h treatment with DMSO, olaparib (Ola, 10 μM), XAV‐939 (XAV, 30 μM) or their combination (O + X). PTEN protein levels are shown in the upper panel. (B) Western blot analysis showing the dose‐dependent effects of JPI‐547 on the expression of Wnt signalling pathway proteins, including phosphorylated β‐catenin, total β‐catenin, Axin1 and Axin2, in Hec‐1A and Ishikawa cells. (C) Protein levels of Wnt signalling components were analysed by Western blot in Ishikawa and Hec‐1A cells (siCont, siPTEN) following treatment with DMSO, olaparib, XAV‐939 or the combination. The results are representative of at least two independent experiments. Relative band intensities normalised to β‐actin are shown above each lane. Molecular weight markers (kDa) are indicated on the right. ND, not detected.

We did not observe a clear association between increased DNA damage and disruption of HR repair involving RAD51 following combined treatment with olaparib and TNKS inhibition in PTEN‐deficient EC cells (Figure S5C). DNA‐PKcs and Ku70/80 are key proteins involved in the non‐homologous end joining (NHEJ) pathway, which mediates the direct re‐ligation of DSBs [28]. We assessed the expression of DNA‐PKcs and Ku70/80 in EC cells with different PTEN statuses following treatment with olaparib and the TNKS inhibitor XAV‐939 (Figure 7A). At baseline, DNA‐PKcs expression was markedly low in Ishikawa cells. Moreover, PTEN knockdown in Hec‐1A cells led to a moderate reduction in DNA‐PKcs levels. Notably, in PTEN‐proficient Hec‐1A cells, combined treatment with olaparib and XAV‐939 led to downregulation of DNA‐PKcs, whereas in PTEN knockdown Hec‐1A cells, DNA‐PKcs levels remained unchanged.

To explore the impact of PARP and TNKS inhibition on the Wnt/β‐catenin signalling pathway, we examined the expression of Wnt‐related proteins in EC cells treated with JPI‐547, olaparib, XAV‐939 or a combination of olaparib and XAV‐939. In Ishikawa cells, JPI‐547 treatment led to an increase in the levels of phosphorylated β‐catenin, whereas no such change was observed in Hec‐1A cells. Notably, Axin1 protein expression increased in both cell lines, while Axin2 levels remained unchanged (Figure 7B). Furthermore, combined treatment with olaparib and XAV‐939 reduced total β‐catenin levels in Ishikawa cells compared to single‐agent treatments, suggesting suppression of canonical Wnt signalling (Figure 7C). However, this pattern was not observed in Hec‐1A cells, regardless of PTEN status. Interestingly, Axin1 protein levels were reduced in PTEN knockdown Hec‐1A cells. Although XAV‐939 alone increased Axin1 expression, the addition of olaparib did not further elevate Axin1 levels in Ishikawa cells. Axin2 levels remained unchanged under all treatment conditions (Figure 7C). Collectively, these data suggest that combined PARP/TNKS inhibition disrupts Wnt signalling more effectively in PTEN‐mutant Ishikawa cells, whereas its impact appears more limited in Hec‐1A cells, irrespective of PTEN expression status.

## 4 Discussion

This study demonstrates the therapeutic efficacy of dual PARP/TNKS inhibition in EC, providing both in vitro and in vivo evidence of its enhanced antitumor activity, particularly in PTEN‐deficient models. JPI‐547 showed the most potent antitumor activity among the PARP inhibitors tested in PTEN‐deficient xenografts. In PTEN‐deficient cells, co‐treatment with olaparib and a TNKS inhibitor enhanced DNA damage and attenuated Wnt/β‐catenin signalling. Loss of PTEN has been proposed as a potential biomarker to predict sensitivity to PARP inhibitors in EC; however, preclinical data have been largely inconsistent and mechanistically inconclusive, limiting its clinical applicability [13, 29]. Although PTEN deficiency has been reported to induce genomic instability in several studies, its association with HR defects remains controversial [9, 12, 27, 30]. These conflicting results underscore the need for alternative strategies or combinatorial approaches to enhance the efficacy of PARP inhibition in PTEN‐deficient EC.

Using representative in vivo EC xenograft models, the present study indicates enhanced sensitivity to multiple PARP inhibitors in PTEN‐deficient Ishikawa tumours, in line with increased vulnerability to DNA repair‐targeting therapies in this molecular context [10, 13, 14]. Notably, JPI‐547 exhibited the most marked tumour growth inhibition among the PARP inhibitors tested in our experimental setting, supporting the therapeutic rationale of concurrent targeting of DNA repair and Wnt signalling pathways [31, 32, 33, 34]. Preliminary clinical trial data from the ongoing phase I study (NCT04335604) have shown promising results with JPI‐547 in advanced solid tumours, particularly in patients harbouring BRCA or HR repair mutations, thereby encouraging further investigation into its translational application for other HR‐deficient cancers, including EC [22]. In addition, we demonstrated the enhanced therapeutic efficacy of dual inhibition of PARP and TNKS and investigated the underlying molecular mechanisms. This was validated through in vitro assays, which confirmed that the combined treatment resulted in synergistic suppression of cell proliferation and long‐term cell survival, particularly in PTEN‐deficient EC cells. Collectively, these findings suggest that co‐targeting TNKS and PARP may represent a rational strategy to overcome the limitations of PARP inhibitor monotherapy in molecularly selected EC populations.

From a mechanistic perspective, comet assay analysis demonstrated that treatment with JPI‐547 or the combination of olaparib and XAV‐939 induced greater DNA damage than either agent alone, with the most pronounced effects in PTEN‐deficient Ishikawa cells (Figure 6). By contrast, γ‐H2AX levels did not differ significantly between the combination treatment and olaparib alone (Figure S5), suggesting that these assays capture distinct aspects of the DNA damage response. While γ‐H2AX primarily reflects early signalling events following DSB formation, the comet assay represents cumulative genomic DNA damage [35]. Accordingly, although DNA damage signalling is activated upon PARP inhibition, the extent of DNA damage induced by concomitant TNKS inhibition may exceed the cellular repair capacity, resulting in persistent DNA damage accumulation. Although the precise mechanisms by which combined PARP and TNKS inhibition enhances DNA damage were not directly elucidated in this study, previous reports have shown that TNKS inhibition can potentiate the effects of DNA‐damaging agents by disrupting mediator of Rap80 interactions and targeting 40 kDa (MERIT40)‐ or mediator of DNA damage checkpoint protein 1 (MDC1)‐dependent repair of DSBs [21, 36]. Given that MDC1 regulates both HR and NHEJ, TNKS inhibition may compromise multiple DNA repair pathways, thus exacerbating DNA damage following PARP inhibition. Consistent with this interpretation, increased nuclear RAD51 signals were observed in PTEN‐deficient EC cells (Figure S5C); nevertheless, dual inhibition of PARP1/2 and TNKS1/2 markedly impaired long‐term cell survival, indicating that enhanced DNA damage ultimately outweighs compensatory repair activity.

The association between PTEN loss and decreased RAD51 expression remains a subject of debate, with inconsistent findings regarding the impact of PTEN deficiency on RAD51 levels and HR activity [12, 13, 14, 27, 37]. Consistent with recent studies, our results in Hec‐1A cells show that PTEN knockdown leads to increased DNA damage and elevated RAD51 levels (Figure 6 and Figure S5), indicating that PTEN loss does not necessarily reduce RAD51 expression [27, 37]. These findings suggest that additional mechanisms beyond HR impairment should be considered to enhance the sensitivity of PARP inhibitors in PTEN‐deficient EC. Nevertheless, the observed increase in DNA damage following PTEN knockdown further supports the role of PTEN in maintaining genomic integrity and regulating the DNA damage response [9, 30, 38, 39].

NHEJ, along with HR, constitutes a major pathway for the repair of DSBs and has been implicated in tumour progression and metastasis in EC [40, 41]. Our analysis of DNA‐PKcs expression revealed distinct patterns across cell lines, with PTEN‐deficient Ishikawa cells showing minimal DNA‐PKcs expression and Hec‐1A cells exhibiting reduced DNA‐PKcs levels following PTEN knockdown (Figure 7A). Consistent with previous reports demonstrating that PTEN positively regulates NHEJ activity [42], loss of DNA‐PKcs function has been associated with impaired replication checkpoint control and increased apoptosis under replication stress [43]. Importantly, inhibition of DNA‐PKcs has been shown to enhance sensitivity to PARP inhibitors [44, 45], suggesting that compromised NHEJ may contribute to the increased responsiveness to dual PARP/TNKS inhibition, including JPI‐547, in PTEN‐deficient EC models.

TNKS enhances Wnt/β‐catenin signalling through the PARylation of Axin1/2, promoting their ubiquitination and subsequent degradation [33, 46]. Our results showed that JPI‐547 suppressed Wnt signalling, as evidenced by increased Axin1 expression in PTEN‐deficient Ishikawa cells (Figure 7B). Likewise, treatment with XAV‐939 also stabilised Axin1 (Figure 7C), consistent with the established mechanism by which TNKS inhibition results in Axin accumulation, subsequent β‐catenin phosphorylation and degradation [33, 47]. Of note, PTEN knockdown reduced Axin1 protein levels in Hec‐1A cells (Figure 7C), indicating an inverse relationship between Axin1 expression and activation of the PI3K/AKT signalling pathway following PTEN loss [48, 49]. Moreover, Ishikawa cells exhibited olaparib‐induced β‐catenin downregulation, with combination treatment showing enhanced suppression compared to single‐agent therapy (Figure 7C). This observation suggests potential crosstalk between PARP inhibition and Wnt signalling, as previous studies have demonstrated that PARP‐1 inhibition can directly suppress β‐catenin expression and its downstream targets, such as c‐Myc and cyclin D1, indicating a possible link between DNA damage repair pathways and Wnt signalling [50].

Despite these promising findings, our study has several limitations. Our experimental system was restricted to three EC cell lines. This may not fully represent the diverse spectrum of PTEN‐mutant ECs, which vary in mutation type and phenotypic behaviour. Although we observed correlations between PTEN deficiency and enhanced efficacy of dual PARP/TNKS inhibition, the mechanistic basis for these associations, particularly the molecular crosstalk between PTEN signalling and TNKS‐mediated pathways in EC, remains incompletely understood.

## 5 Conclusions

Our study highlights the therapeutic potential of JPI‐547 in targeting PTEN‐deficient EC by promoting DNA damage associated with impaired NHEJ and inhibiting Wnt/β‐catenin signalling. Combined inhibition of PARP and TNKS provides a multifaceted approach to overcome compensatory DNA repair mechanisms that contribute to resistance against single‐agent therapies.

## Author Contributions

**Tae Won Kim:** funding acquisition, project administration. **Hyerim Eum:** writing – original draft, investigation, writing – review and editing. **Shin‐Wha Lee:** funding acquisition, project administration, supervision, conceptualization. **Sung Wan Kang:** writing – original draft, investigation, conceptualization, writing – review and editing, formal analysis, visualization. **Min‐Seo Lee:** investigation. **Ji‐Young Lee:** investigation. **Yong‐Man Kim:** project administration, conceptualization.

## Funding

This research was supported by a grant from the Korea Health Technology R&D Project through the Korea Health Industry Development Institute (KHIDI), funded by the Ministry of Health & Welfare, Republic of Korea (grant number: RS‐2023‐00261982). This study was also supported by a grant (grant number: 2024IP0090) from the Asan Institute for Life Sciences, Asan Medical Center, Seoul, Korea.

## Conflicts of Interest

The authors declare no conflicts of interest.

## Supporting information


**Data S1:** Supplementary methods. Detailed experimental procedures and additional methodological information.

**Figure S1:** Body weight changes in Hec‐1A and Ishikawa xenograft models following treatment with PARP inhibitors. Body weight was monitored as an indicator of drug‐induced toxicity or adverse effects in mice bearing (A) Hec‐1A and (B) Ishikawa xenografts. Mice received the indicated treatments, and body weight was measured at regular intervals throughout the treatment period.

**Figure S2:** Cell viability of EC cells following treatment with JPI‐547, olaparib and XAV‐939. A summary table of IC_50_ values shows quantitative differences in drug response across the cell lines. Cell viability was assessed using the luminescent CellTiter‐Glo assay in three EC cell lines (Hec‐1A, Hec‐1B and Ishikawa) treated with increasing concentrations of JPI‐547, olaparib or XAV‐939 for 120 h. Cell viability was normalised to DMSO‐treated controls, and dose–response curves were generated to calculate GI_50_ values.

**Figure S3:** Induction of apoptosis by olaparib and JPI‐547 in EC cells. Hec‐1A and Ishikawa cells were treated with DMSO, olaparib (10 μM) or JPI‐547 (10 μM) for 72 h. (A) Apoptotic cell populations were assessed by flow cytometry using Annexin V‐FITC and PI staining. Quadrant analysis was used to distinguish viable cells (Annexin V^−^/PI^−^), early apoptotic cells (Annexin V^+^/PI^−^), late apoptotic cells (Annexin V^+^/PI^+^) and necrotic cells (Annexin V^−^/PI^+^). (B) Quantification results are presented as mean ± SD from three independent experiments. Statistical significance was determined using an unpaired two‐tailed Student's *t*‐test (*p < 0.05, **p < 0.01, n.s. = not significant). Asterisks (*) indicate statistical significance compared with the DMSO control.

**Figure S4:** Clonogenic assays demonstrating the long‐term anti‐proliferative effects of JPI‐547, olaparib and XAV‐939 in EC cell lines. The IC_50_ summary table highlights differential drug sensitivity across the cell lines. All compounds elicited dose‐dependent inhibition of colony formation. Colony numbers were normalised to DMSO‐treated controls, and dose–response curves were generated to calculate IC_50_ values.

**Figure S5:** Effects of combined PARP and TNKS inhibition on DNA damage and RAD51 expression in EC cells. (A) Representative IF images showing γ‐H2AX (red) and RAD51 (green) staining in Hec‐1A cells transfected with siPTEN or control siRNA (siCont), and in Ishikawa cells treated for 48 h with olaparib (10 μM), XAV‐939 (30 μM), their combination or JPI‐547 (10 μM). Nuclei were counterstained with DAPI (blue). Quantification of (B) γ‐H2AX‐ and (C) RAD51‐positive cells indicates treatment‐dependent alterations in DNA damage and nuclear RAD51 signal. γ‐H2AX foci were quantified as the percentage of cells containing ≥ 5 nuclear γ‐H2AX foci. RAD51 was quantified as the percentage of cells exhibiting nuclear RAD51 signal above background levels. Statistical significance was determined by one‐way ANOVA followed by Tukey's post hoc test (**p < 0.01, ***p < 0.001, #*p* < 0.05, ###*p* < 0.001). Asterisks (*) indicate statistical significance compared with the control.

**Table S1:** Original cell viability data used for CI analysis in Figure 5A.

## Data Availability

Data are available from the corresponding author upon reasonable request.
