# Supplementary material for: Dual PARP/Tankyrase Inhibition Enhances Antitumor Efficacy in PTEN‐Deficient Endometrial Cancer
Source: J Cell Mol Med. 2026 Jun 12;30(11):e71242. doi: 10.1111/jcmm.71242 (PMC13263240; doi:10.1111/jcmm.71242)
Supplement: Supplementary file 1 — Data S1: Supplementary methods. Detailed experimental procedures and additional methodological information. [file JCMM-30-e71242-s003.docx]

**Supplementary Methods**

**Drugs and reagents**

Dimethyl sulfoxide (DMSO) was obtained from Duchefa Biochemie (Haarlem, Netherlands), and Cremophor EL was purchased from Sigma-Aldrich. The PARP inhibitors olaparib, niraparib, and talazoparib were purchased from ChemScene (Monmouth Junction, NJ, USA). JPI-547 was kindly provided by Onconic Therapeutics Inc. (Seoul, Korea). All compounds were dissolved in DMSO and stored as aliquots at -20 ℃. For oral administration in mice, drug formulations were prepared using 10% DMSO (stock solution), 10% Cremophor EL, and 80% distilled water.

**Annexin V/PI apoptosis assay**

Apoptotic cell death was evaluated using the Annexin V-FITC apoptosis detection kit (Koma Biotech, Seoul, Korea) according to the manufacturer’s protocol. Hec-1A and Ishikawa cells were treated with olaparib or JPI-547 for 72 hours. Following treatment, cells were trypsinized, collected, and washed twice with PBS. Subsequently, cells were stained with FITC-conjugated Annexin V and propidium iodide (PI). Flow cytometry analysis was performed using a FACS Canto II (BD Biosciences, Franklin Lakes, NJ, USA), and the data were processed with FlowJo software (TreeStar, Woodburn, OR, USA).

**Immunofluorescence staining analysis of γH2AX and RAD51**

Cells were seeded onto 20-mm immunofluorescence culture dishes (NEST Biotechnology, Wuxi, China) and treated with compounds for 24 hours. After treatment, cells were fixed with 4% paraformaldehyde for 30 minutes at RT, followed by permeabilization with 0.5% Triton X-100 in PBS for 30 minutes at RT. Subsequently, nonspecific binding was blocked with 1% bovine serum albumin (BSA) in Tris-buffered saline with Tween 20 (TBST) for 1 hour at RT. Cells were then incubated overnight at 4 °C with Alexa Fluor 647 anti-γ-H2AX (Abcam) and Alexa Fluor 488 anti-Rad51 (Abcam). After three washes with TBST, coverslips were mounted using a DAPI-containing mounting medium (Abcam). Images were captured using an LSM 780 confocal microscope (Carl Zeiss, Oberkochen, Germany). Quantitative analysis was performed using ImageJ software. For γ-H2AX, cells exhibiting ≥5 distinct nuclear γ-H2AX foci were defined as γ-H2AX-positive cells, and the percentage of γ-H2AX-positive cells was calculated. For RAD51, because staining was predominantly diffuse within the nucleus and discrete foci were not consistently observed, quantification was based on nuclear signal intensity rather than foci counts. Cells exhibiting nuclear RAD51 signal above background levels were defined as RAD51-positive, and the percentage of RAD51-positive cells was calculated.
