# Supplementary material for: Dual PARP/Tankyrase Inhibition Enhances Antitumor Efficacy in PTEN‐Deficient Endometrial Cancer
Source: J Cell Mol Med. 2026 Jun 12;30(11):e71242. doi: 10.1111/jcmm.71242 (PMC13263240; doi:10.1111/jcmm.71242)
Supplement: Supplementary file 2 — Figure S1: Body weight changes in Hec‐1A and Ishikawa xenograft models following treatment with PARP inhibitors. Body weight was monitored as an indicator of drug‐induced toxicity or adverse effects in mice bearing (A) Hec‐1A and (B) Ishikawa xenografts. Mice received the indicated treatments, and body weight was measured at regular intervals throughout the treatment period. [file JCMM-30-e71242-s007.docx]

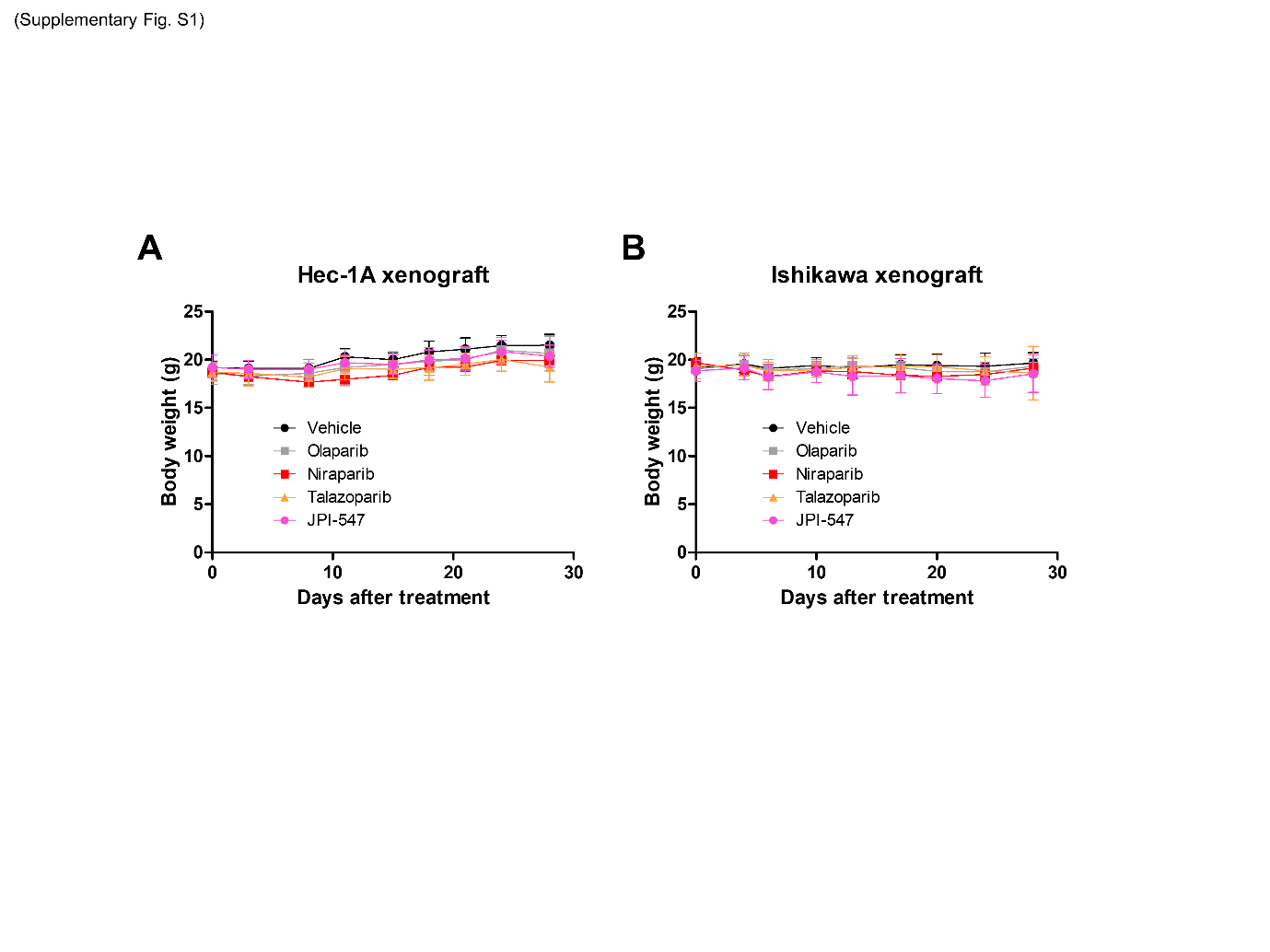
**Supplementary Fig. S1. Body weight changes in Hec-1A and Ishikawa xenograft models following treatment with PARP inhibitors.** Body weight was monitored as an indicator of drug-induced toxicity or adverse effects in mice bearing (A) Hec-1A and (B) Ishikawa xenografts. Mice received the indicated treatments, and body weight was measured at regular intervals throughout the treatment period.
