# Supplementary material for: Dual PARP/Tankyrase Inhibition Enhances Antitumor Efficacy in PTEN‐Deficient Endometrial Cancer
Source: J Cell Mol Med. 2026 Jun 12;30(11):e71242. doi: 10.1111/jcmm.71242 (PMC13263240; doi:10.1111/jcmm.71242)
Supplement: Supplementary file 3 — Figure S2: Cell viability of EC cells following treatment with JPI‐547, olaparib and XAV‐939. A summary table of IC50 values shows quantitative differences in drug response across the cell lines. Cell viability was assessed using the luminescent CellTiter‐Glo assay in three EC cell lines (Hec‐1A, Hec‐1B and Ishikawa) treated with increasing concentrations of JPI‐547, olaparib or XAV‐939 for 120 h. Cell viability was normalised to DMSO‐treated controls, and dose–response curves were generated to calculate GI50 values. [file JCMM-30-e71242-s004.docx]

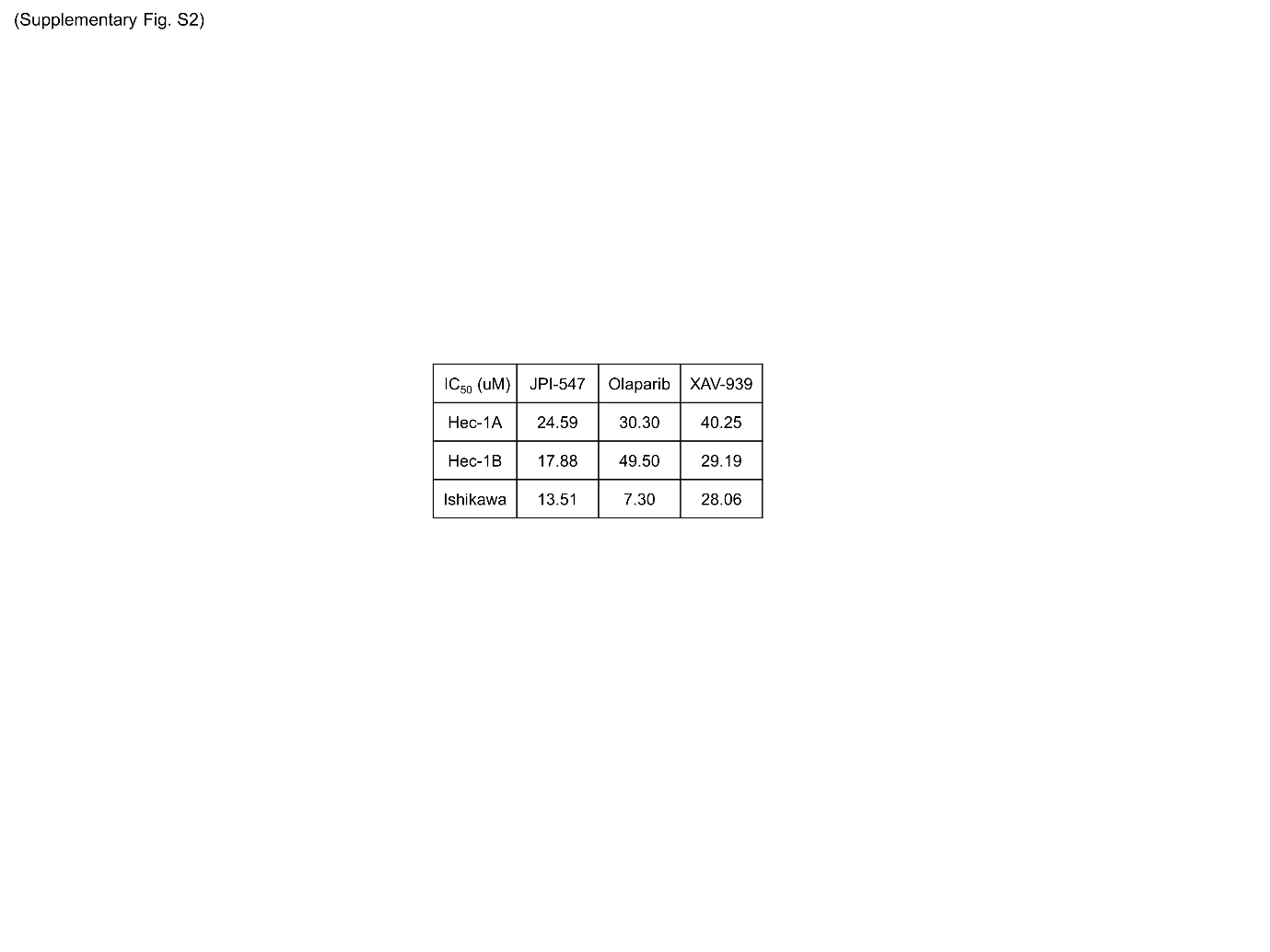


**Supplementary Fig. S2. Cell viability of EC cells following treatment with JPI-547, olaparib, and XAV-939.** A summary table of IC_50_ values shows quantitative differences in drug response across the cell lines. Cell viability was assessed using the luminescent CellTiter-Glo assay in three EC cell lines (Hec-1A, Hec-1B, and Ishikawa) treated with increasing concentrations of JPI-547, olaparib, or XAV-939 for 120 hours. Cell viability was normalized to DMSO-treated controls, and dose-response curves were generated to calculate GI_50_ values.
