# Supplementary material for: Dual PARP/Tankyrase Inhibition Enhances Antitumor Efficacy in PTEN‐Deficient Endometrial Cancer
Source: J Cell Mol Med. 2026 Jun 12;30(11):e71242. doi: 10.1111/jcmm.71242 (PMC13263240; doi:10.1111/jcmm.71242)
Supplement: Supplementary file 4 — Figure S3: Induction of apoptosis by olaparib and JPI‐547 in EC cells. Hec‐1A and Ishikawa cells were treated with DMSO, olaparib (10 μM) or JPI‐547 (10 μM) for 72 h. (A) Apoptotic cell populations were assessed by flow cytometry using Annexin V‐FITC and PI staining. Quadrant analysis was used to distinguish viable cells (Annexin V−/PI−), early apoptotic cells (Annexin V+/PI−), late apoptotic cells (Annexin V+/PI+) and necrotic cells (Annexin V−/PI+). (B) Quantification results are presented as mean ± SD from three independent experiments. Statistical significance was determined using an unpaired two‐tailed Student's t‐test (*p < 0.05, **p < 0.01, n.s. = not significant). Asterisks (*) indicate statistical significance compared with the DMSO control. [file JCMM-30-e71242-s005.docx]

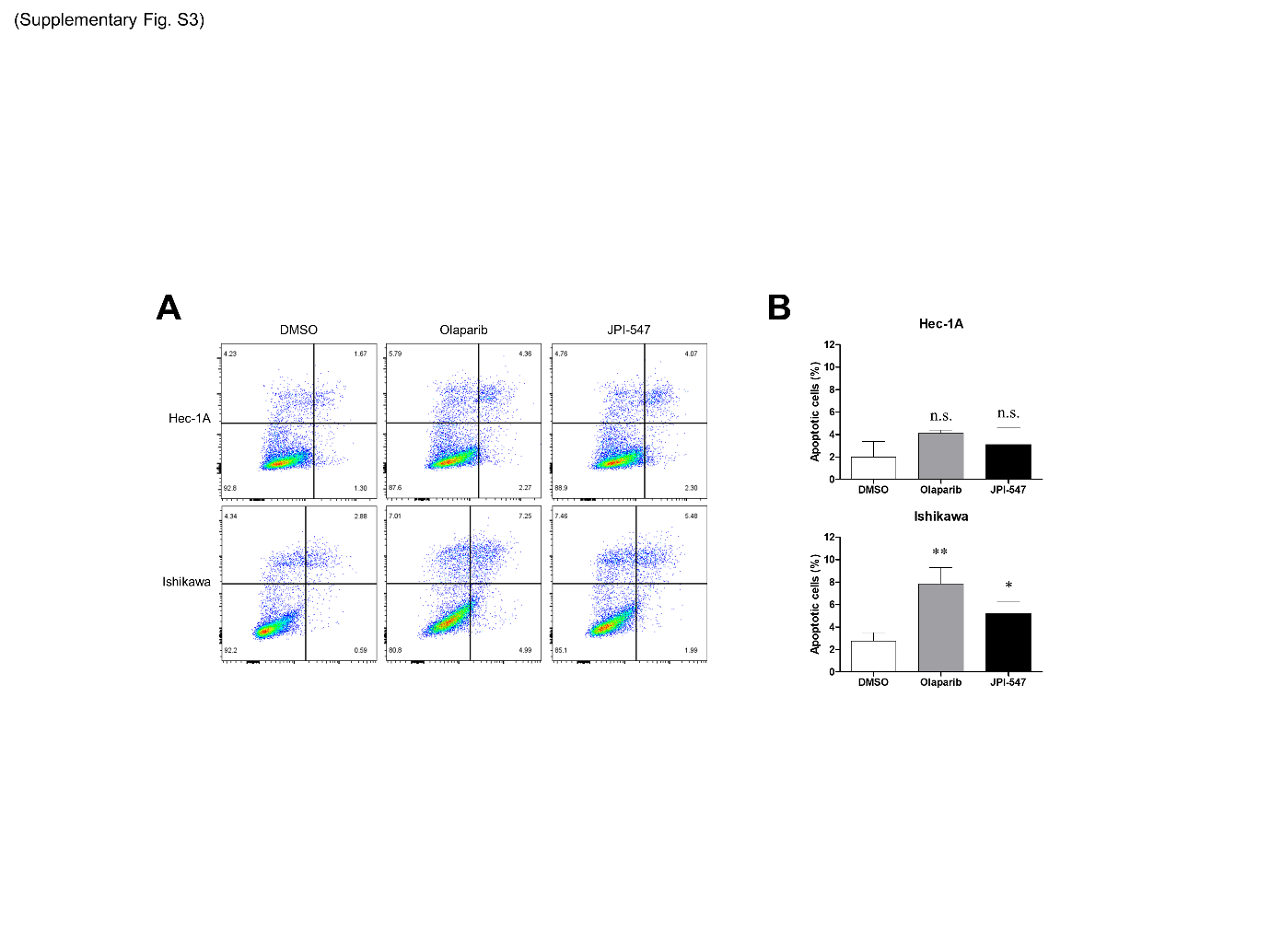


**Supplementary Fig. S3. Induction of apoptosis by olaparib and JPI-547 in EC cells.** Hec-1A and Ishikawa cells were treated with DMSO, olaparib (10 μM), or JPI-547 (10 μM) for 72 hours. (A) Apoptotic cell populations were assessed by flow cytometry using Annexin V-FITC and PI staining. Quadrant analysis was used to distinguish viable cells (Annexin V⁻/PI⁻), early apoptotic cells (Annexin V⁺/PI⁻), late apoptotic cells (Annexin V⁺/PI⁺), and necrotic cells (Annexin V⁻/PI⁺). (B) Quantification results are presented as mean ± SD from three independent experiments. Statistical significance was determined using an unpaired two-tailed Student’s t-test (*P < 0.05, **P < 0.01, n.s. = not significant). Asterisks (*) indicate statistical significance compared with the DMSO control.
