# Supplementary material for: Dual PARP/Tankyrase Inhibition Enhances Antitumor Efficacy in PTEN‐Deficient Endometrial Cancer
Source: J Cell Mol Med. 2026 Jun 12;30(11):e71242. doi: 10.1111/jcmm.71242 (PMC13263240; doi:10.1111/jcmm.71242)
Supplement: Supplementary file 5 — Figure S4: Clonogenic assays demonstrating the long‐term anti‐proliferative effects of JPI‐547, olaparib and XAV‐939 in EC cell lines. The IC50 summary table highlights differential drug sensitivity across the cell lines. All compounds elicited dose‐dependent inhibition of colony formation. Colony numbers were normalised to DMSO‐treated controls, and dose–response curves were generated to calculate IC50 values. [file JCMM-30-e71242-s002.docx]

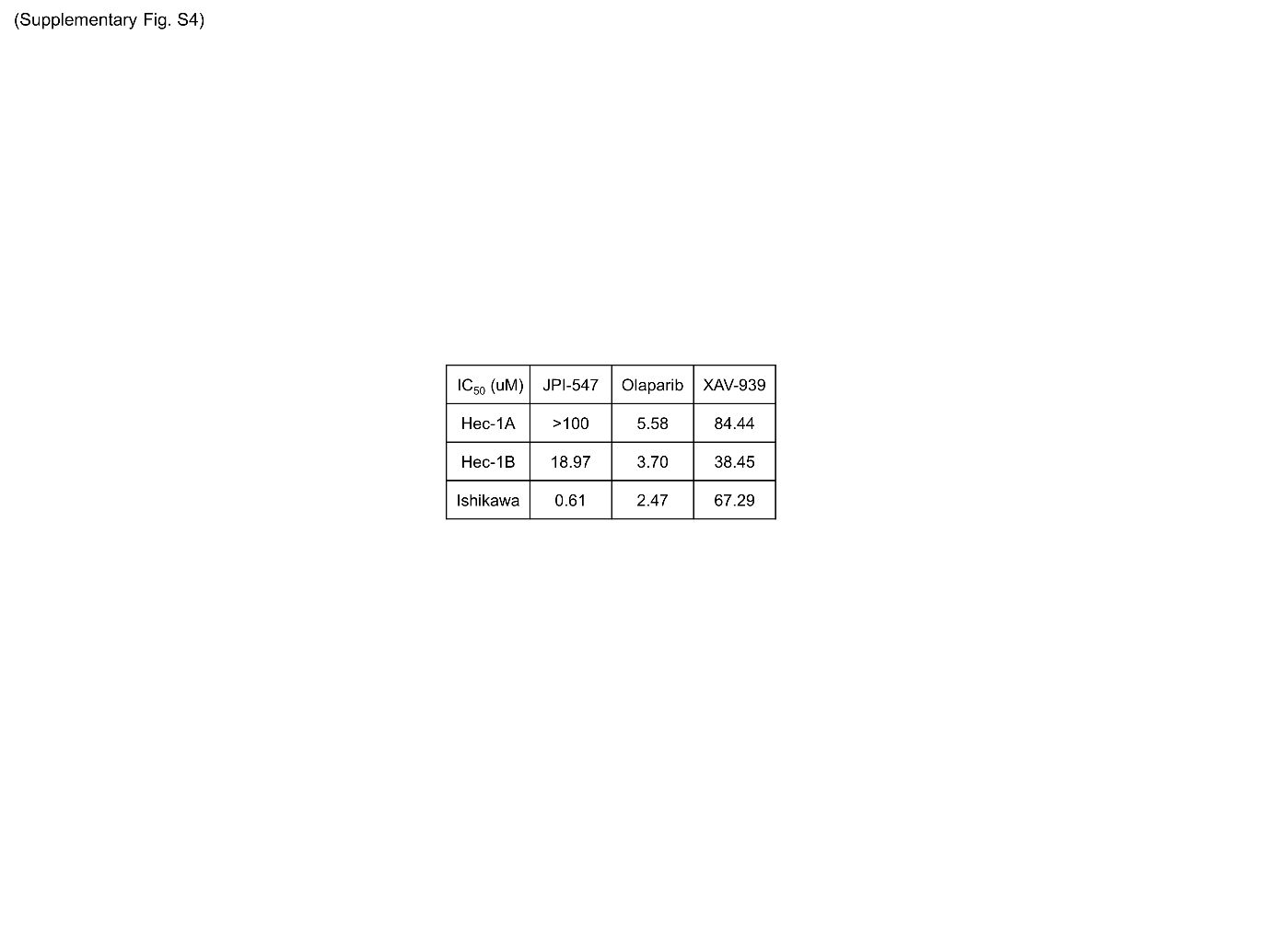


**Supplementary Fig. S4. Clonogenic assays demonstrating the long-term anti-proliferative effects of JPI-547, olaparib, and XAV-939 in EC cell lines.** The IC_50_ summary table highlights differential drug sensitivity across the cell lines. All compounds elicited dose-dependent inhibition of colony formation. Colony numbers were normalized to DMSO-treated controls, and dose-response curves were generated to calculate IC_50_ values.
