# Supplementary material for: Dual PARP/Tankyrase Inhibition Enhances Antitumor Efficacy in PTEN‐Deficient Endometrial Cancer
Source: J Cell Mol Med. 2026 Jun 12;30(11):e71242. doi: 10.1111/jcmm.71242 (PMC13263240; doi:10.1111/jcmm.71242)
Supplement: Supplementary file 6 — Figure S5: Effects of combined PARP and TNKS inhibition on DNA damage and RAD51 expression in EC cells. (A) Representative IF images showing γ‐H2AX (red) and RAD51 (green) staining in Hec‐1A cells transfected with siPTEN or control siRNA (siCont), and in Ishikawa cells treated for 48 h with olaparib (10 μM), XAV‐939 (30 μM), their combination or JPI‐547 (10 μM). Nuclei were counterstained with DAPI (blue). Quantification of (B) γ‐H2AX‐ and (C) RAD51‐positive cells indicates treatment‐dependent alterations in DNA damage and nuclear RAD51 signal. γ‐H2AX foci were quantified as the percentage of cells containing ≥ 5 nuclear γ‐H2AX foci. RAD51 was quantified as the percentage of cells exhibiting nuclear RAD51 signal above background levels. Statistical significance was determined by one‐way ANOVA followed by Tukey's post hoc test (**p < 0.01, ***p < 0.001, #p < 0.05, ###p < 0.001). Asterisks (*) indicate statistical significance compared with the control. [file JCMM-30-e71242-s006.docx]

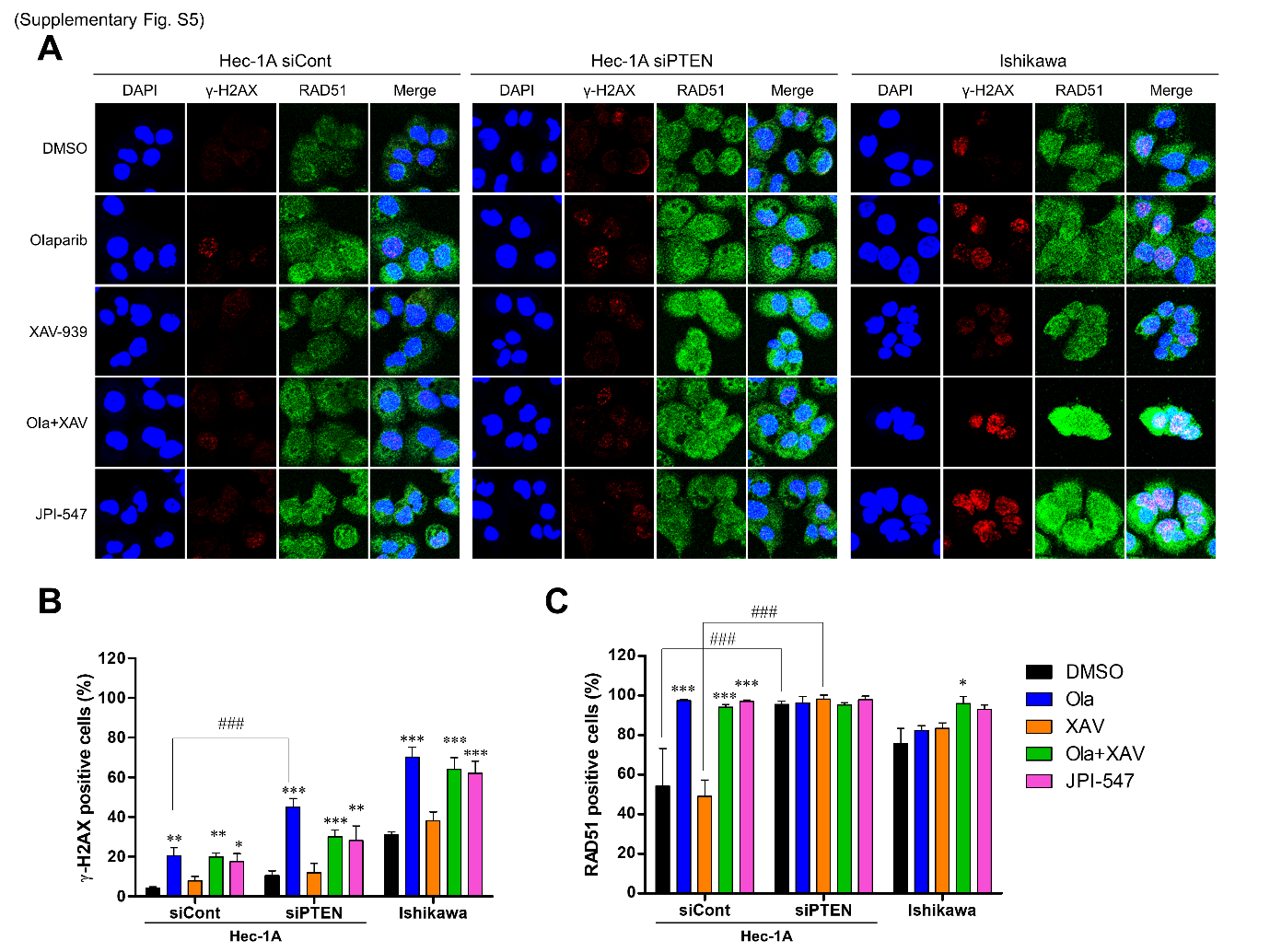


**Supplementary Fig. S5. Effects of combined PARP and TNKS inhibition on DNA damage and RAD51 expression in EC cells.** (A) Representative IF images showing γ-H2AX (red) and RAD51 (green) staining in Hec-1A cells transfected with siPTEN or control siRNA (siCont), and in Ishikawa cells treated for 48 hours with olaparib (10 μM), XAV-939 (30 μM), their combination, or JPI-547 (10 μM). Nuclei were counterstained with DAPI (blue). Quantification of (B) γ-H2AX- and (C) RAD51-positive cells indicates treatment-dependent alterations in DNA damage and nuclear RAD51 signal. γ-H2AX foci were quantified as the percentage of cells containing ≥5 nuclear γ-H2AX foci. RAD51 was quantified as the percentage of cells exhibiting nuclear RAD51 signal above background levels. Statistical significance was determined by one-way ANOVA followed by Tukey’s post hoc test (**P < 0.01, ***P < 0.001, #P < 0.05, ###P < 0.001). Asterisks (*) indicate statistical significance compared with the control.
