# Supplementary material for: Dual PARP/Tankyrase Inhibition Enhances Antitumor Efficacy in PTEN‐Deficient Endometrial Cancer
Source: J Cell Mol Med. 2026 Jun 12;30(11):e71242. doi: 10.1111/jcmm.71242 (PMC13263240; doi:10.1111/jcmm.71242)
Supplement: Supplementary file 7 — Table S1: Original cell viability data used for CI analysis in Figure 5A. [file JCMM-30-e71242-s001.docx]

| Supplementary Table S1. Original cell viability data used for CI analysis in Figure 5A. | | | | | |
| --- | --- | --- | --- | --- | --- |
| Cell line | Olaparib  (μM) | XAV-939  (μM) | Viability  (%, Mean±SD) | Fraction affected (Fa, %) | Combination Index (CI) |
| Hec-1A | 0 | 0 | 100.00±1.56 | 0.00 | - |
| Hec-1A | 2.5 | 0 | 96.60±3.08 | 3.40 | - |
| Hec-1A | 7.4 | 0 | 78.43±2.91 | 21.57 | - |
| Hec-1A | 22.2 | 0 | 62.94±1.52 | 37.06 | - |
| Hec-1A | 66.6 | 0 | 44.11±3.10 | 55.89 | - |
| Hec-1A | 200 | 0 | 16.31±1.29 | 83.69 | - |
| Hec-1A | 0 | 12.5 | 89.70±3.38 | 10.30 | - |
| Hec-1A | 2.5 | 12.5 | 79.53±2.51 | 20.47 | 0.70 |
| Hec-1A | 7.4 | 12.5 | 68.23±1.73 | 31.77 | 0.61 |
| Hec-1A | 22.2 | 12.5 | 54.25±1.63 | 45.75 | 0.75 |
| Hec-1A | 66.6 | 12.5 | 38.42±0.47 | 61.58 | 1.07 |
| Hec-1A | 200 | 12.5 | 15.31±1.69 | 84.69 | 0.95 |
| Hec-1A | 0 | 25 | 76.16±1.55 | 23.84 | - |
| Hec-1A | 2.5 | 25 | 70.25±4.37 | 29.75 | 0.68 |
| Hec-1A | 7.4 | 25 | 60.91±2.88 | 39.09 | 0.60 |
| Hec-1A | 22.2 | 25 | 48.79±1.76 | 51.21 | 0.69 |
| Hec-1A | 66.6 | 25 | 33.55±1.83 | 66.45 | 0.92 |
| Hec-1A | 200 | 25 | 12.65±0.40 | 87.35 | 0.78 |
| Hec-1A | 0 | 50 | 65.97±2.52 | 34.03 | - |
| Hec-1A | 2.5 | 50 | 62.78±1.55 | 37.22 | 0.83 |
| Hec-1A | 7.4 | 50 | 56.08±2.14 | 43.92 | 0.75 |
| Hec-1A | 22.2 | 50 | 43.63±1.09 | 56.37 | 0.71 |
| Hec-1A | 66.6 | 50 | 28.18±1.03 | 71.82 | 0.78 |
| Hec-1A | 200 | 50 | 11.16±0.75 | 88.84 | 0.70 |
| Hec-1A | 0 | 100 | 59.03±3.86 | 40.97 | - |
| Hec-1A | 2.5 | 100 | 52.72±2.57 | 47.28 | 0.96 |
| Hec-1A | 7.4 | 100 | 43.58±0.93 | 56.42 | 0.72 |
| Hec-1A | 22.2 | 100 | 34.00±5.46 | 66.00 | 0.64 |
| Hec-1A | 66.6 | 100 | 20.53±1.49 | 79.47 | 0.60 |
| Hec-1A | 200 | 100 | 8.41±1.31 | 91.59 | 0.54 |
| Hec-1B | 0 | 0 | 100.00±1.95 | 0.00 | - |
| Hec-1B | 2.5 | 0 | 108.64±4.55 | -8.64 | - |
| Hec-1B | 7.4 | 0 | 99.34±2.43 | 0.66 | - |
| Hec-1 B | 22.2 | 0 | 78.84±1.45 | 21.16 | - |
| Hec-1 B | 66.6 | 0 | 53.75±2.01 | 46.25 | - |
| Hec-1 B | 200 | 0 | 31.54±1.23 | 68.46 | - |
| Hec-1 B | 0 | 12.5 | 88.12±4.05 | 11.88 | - |
| Hec-1 B | 2.5 | 12.5 | 91.91±1.16 | 8.09 | 1.88 |
| Hec-1 B | 7.4 | 12.5 | 85.18±1.43 | 14.82 | 1.03 |
| Hec-1 B | 22.2 | 12.5 | 65.38±2.24 | 34.62 | 0.58 |
| Hec-1B | 66.6 | 12.5 | 44.32±2.14 | 55.68 | 0.73 |
| Hec-1B | 200 | 12.5 | 26.12±1.66 | 73.88 | 1.23 |
| Hec-1B | 0 | 25 | 76.79±1.55 | 23.21 | - |
| Hec-1 B | 2.5 | 25 | 79.49±1.86 | 20.51 | 1.06 |
| Hec-1 B | 7.4 | 25 | 73.13±2.10 | 26.87 | 0.81 |
| Hec-1 B | 22.2 | 25 | 59.36±1.11 | 40.64 | 0.63 |
| Hec-1 B | 66.6 | 25 | 41.23±1.30 | 58.77 | 0.74 |
| Hec-1 B | 200 | 25 | 23.87±1.48 | 76.13 | 1.17 |
| Hec-1 B | 0 | 50 | 71.37±3.30 | 28.63 | - |
| Hec-1 B | 2.5 | 50 | 71.58±2.17 | 28.42 | 1.25 |
| Hec-1B | 7.4 | 50 | 63.72±1.47 | 36.28 | 0.91 |
| Hec-1B | 22.2 | 50 | 52.03±0.55 | 47.97 | 0.71 |
| Hec-1B | 66.6 | 50 | 38.60±1.11 | 61.40 | 0.80 |
| Hec-1 B | 200 | 50 | 23.08±0.88 | 76.92 | 1.19 |
| Hec-1 B | 0 | 100 | 53.14±3.46 | 46.86 | - |
| Hec-1 B | 2.5 | 100 | 53.64±0.89 | 46.36 | 1.00 |
| Hec-1 B | 7.4 | 100 | 48.83±2.53 | 51.17 | 0.85 |
| Hec-1 B | 22.2 | 100 | 42.93±3.41 | 57.07 | 0.79 |
| Hec-1 B | 66.6 | 100 | 32.37±1.32 | 67.63 | 0.83 |
| Hec-1 B | 200 | 100 | 19.73±0.28 | 80.27 | 1.13 |
| Ishikawa | 0 | 0 | 100.00±6.30 | 0.00 | - |
| Ishikawa | 2.5 | 0 | 86.20±1.56 | 13.80 | - |
| Ishikawa | 7.4 | 0 | 58.96±1.22 | 41.04 | - |
| Ishikawa | 22.2 | 0 | 36.75±0.82 | 63.25 | - |
| Ishikawa | 66.6 | 0 | 26.62±3.95 | 73.38 | - |
| Ishikawa | 200 | 0 | 17.99±2.25 | 82.01 | - |
| Ishikawa | 0 | 12.5 | 86.29±2.61 | 13.71 | - |
| Ishikawa | 2.5 | 12.5 | 65.76±1.34 | 34.24 | 0.56 |
| Ishikawa | 7.4 | 12.5 | 42.26±3.71 | 57.74 | 0.34 |
| Ishikawa | 22.2 | 12.5 | 26.96±1.57 | 73.04 | 0.35 |
| Ishikawa | 66.6 | 12.5 | 24.94±1.91 | 75.06 | 0.88 |
| Ishikawa | 200 | 12.5 | 13.65±0.26 | 86.35 | 0.94 |
| Ishikawa | 0 | 25 | 80.42±5.64 | 19.58 | - |
| Ishikawa | 2.5 | 25 | 55.29±3.29 | 44.71 | 0.44 |
| Ishikawa | 7.4 | 25 | 36.85±1.32 | 63.15 | 0.30 |
| Ishikawa | 22.2 | 25 | 23.77±1.61 | 76.23 | 0.30 |
| Ishikawa | 66.6 | 25 | 21.09±0.56 | 78.91 | 0.67 |
| Ishikawa | 200 | 25 | 11.72±1.61 | 88.28 | 0.75 |
| Ishikawa | 0 | 50 | 69.33±5.51 | 30.67 | - |
| Ishikawa | 2.5 | 50 | 50.44±1.84 | 49.56 | 0.53 |
| Ishikawa | 7.4 | 50 | 34.27±0.82 | 65.73 | 0.34 |
| Ishikawa | 22.2 | 50 | 23.03±0.50 | 76.97 | 0.33 |
| Ishikawa | 66.6 | 50 | 19.38±0.28 | 80.62 | 0.61 |
| Ishikawa | 200 | 50 | 8.77±0.18 | 91.23 | 0.49 |
| Ishikawa | 0 | 100 | 54.42±0.86 | 45.58 | - |
| Ishikawa | 2.5 | 100 | 38.23±1.36 | 61.77 | 0.49 |
| Ishikawa | 7.4 | 100 | 23.55±0.77 | 76.45 | 0.26 |
| Ishikawa | 22.2 | 100 | 14.34±1.22 | 85.66 | 0.19 |
| Ishikawa | 66.6 | 100 | 11.81±1.14 | 88.19 | 0.31 |
| Ishikawa | 200 | 100 | 5.17±0.44 | 94.83 | 0.24 |
| CI values were interpreted as follows: CI < 0.1, very strong synergism; 0.1-0.3, strong synergism; 0.3-0.7, synergism; 0.7-0.85, moderate synergism; and 0.85-0.90, slight synergism. | | | | | |
